# Aberrant resting-state co-activation network dynamics in major depressive disorder

**DOI:** 10.1038/s41398-023-02722-w

**Published:** 2024-01-03

**Authors:** Ziqi An, Kai Tang, Yuanyao Xie, Chuanjun Tong, Jiaming Liu, Quan Tao, DIRECT Consortium, Chao-Gan Yan, Xiao Chen, Li-Ping Cao, Wei Chen, Yu-Qi Cheng, Yi-Ru Fang, Qi-Yong Gong, Wen-Bin Guo, Li Kuang, Bao-Juan Li, Tao Li, Yan-Song Liu, Zhe-Ning Liu, Jian-Ping Lu, Qing-Hua Luo, Hua-Qing Meng, Dai-Hui Peng, Jiang Qiu, Yue-Di Shen, Tian-Mei Si, Yan-Qing Tang, Chuan-Yue Wang, Fei Wang, Hua-Ning Wang, Kai Wang, Xiang Wang, Ying Wang, Xiao-Ping Wu, Chun-Ming Xie, Guang-Rong Xie, Peng Xie, Xiu-Feng Xu, Hong Yang, Jian Yang, Shu-Qiao Yao, Yong-Qiang Yu, Yong-Gui Yuan, Ke-Rang Zhang, Wei Zhang, Zhi-Jun Zhang, Jun-Juan Zhu, Xi-Nian Zuo, Jing-Ping Zhao, Yu-Feng Zang, Yanqiu Feng

**Affiliations:** 1 School of Biomedical Engineering, Southern Medical University, Guangzhou, China; 2 Institute of Neuroscience, CAS Key Laboratory of Primate Neurobiology, Center for Excellence in Brain Science and Intelligence Technology, Chinese Academy of Sciences, Shanghai, China; 3 Department of Radiology, Shunde Hospital, Southern Medical University (The First People’s Hospital of Shunde), Foshan, China; 4 Department of Rehabilitation Medicine, Zhujiang Hospital, Southern Medical University, Guangzhou, China; 5 Key Laboratory of Mental Health of the Ministry of Education, Guangdong-Hong Kong-Macao Greater Bay Area Center for Brain Science and Brain-Inspired Intelligence, Guangdong Province Key Laboratory of Psychiatric Disorders, Department of Neurobiology, School of Basic Medical Sciences, Southern Medical University, Guangzhou, 510515 China; 6 CAS Key Laboratory of Behavioral Science, Institute of Psychology, Chinese Academy of Sciences, Beijing, 100101 China; 7 International Big-Data Center for Depression Research, Chinese Academy of Sciences, 100101 Beijing, China; 8 Magnetic Resonance Imaging Research Center, Institute of Psychology, Chinese Academy of Sciences, 100101 Beijing, China; 9 Department of Psychology, University of Chinese Academy of Sciences, 100049 Beijing, China; 10 Sino-Danish College, University of Chinese Academy of Sciences, 101408 Beijing, China; 11 Sino-Danish Center for Education and Research, Graduate University of Chinese Academy of Sciences, 101408 Beijing, China; 12 Affiliated Brain Hospital of Guangzhou Medical University, Guangzhou, 510370 China; 13 Department of Psychiatry, Sir Run Run Shaw Hospital, Zhejiang University School of Medicine, Hangzhou, 310020 Zhejiang China; 14 Department of Psychiatry, First Affiliated Hospital of Kunming Medical University, Kunming, 650032 Yunnan China; 15 Shanghai Mental Health Center, Shanghai Jiao Tong University School of Medicine, Shanghai, 200030 China; 16 Huaxi MR Research Center (HMRRC), Department of Radiology, West China Hospital of Sichuan University, Chengdu, Sichuan 610044 China; 17 Research Unit of Psychoradiology, Chinese Academy of Medical Sciences, Chengdu, 610052 Sichuan China; 18 Department of Psychiatry, and National Clinical Research Center for Mental Disorders, The Second Xiangya Hospital of Central South University, Changsha, 410011 Hunan China; 19 Department of Psychiatry, The First Affiliated Hospital of Chongqing Medical University, Chongqing, 400042 China; 20 Xijing Hospital of Air Force Military Medical University, Xi’an, Shaanxi 710032 China; 21 Affiliated Mental Health Center & Hangzhou Seventh People’s Hospital, Zhejiang University School of Medicine, Hangzhou, 310063 Zhejiang China; 22 Mental Health Center and Psychiatric Laboratory, West China Hospital of Sichuan University, Chengdu, 610044 Sichuan China; 23 Department of Clinical Psychology, Suzhou Psychiatric Hospital, The Affiliated Guangji Hospital of Soochow University, Suzhou, 215003 Jiangsu China; 24 Shenzhen Kangning Hospital, Shenzhen, Guangzhou, 518020 China; 25 Faculty of Psychology, Southwest University, Chongqing, 400715 China; 26 Department of Diagnostics, Affiliated Hospital, Hangzhou Normal University Medical School, Hangzhou, 311121 Zhejiang China; 27 National Clinical Research Center for Mental Disorders (Peking University Sixth Hospital) & Key Laboratory of Mental Health, Ministry of Health (Peking University), 100191 Beijing, China; 28 Department of Psychiatry, First Affiliated Hospital, China Medical University, Shenyang, 110122 Liaoning China; 29 Beijing Anding Hospital, Capital Medical University, 100120 Beijing, China; 30 Early Intervention Unit, Department of Psychiatry, Affiliated Nanjing Brain Hospital, Nanjing Medical University, Nanjing, 210024 China; 31 Department of Neurology, The First Affiliated Hospital of Anhui Medical University, Hefei, 230022 Anhui China; 32 The First Affiliated Hospital of Jinan University, Guangzhou, Guangdong, 250024 China; 33 Xi’an Central Hospital, Xi’an, 710004 Shaanxi China; 34 Department of Neurology, Affiliated ZhongDa Hospital of Southeast University, Nanjing, 210009 Jiangsu China; 35 Institute of Neuroscience, Chongqing Medical University, Chongqing, 400016 China; 36 Chongqing Key Laboratory of Neurobiology, Chongqing, 400000 China; 37 Department of Neurology, The First Affiliated Hospital of Chongqing Medical University, Chongqing, 400042 China; 38 Department of Radiology, The First Affiliated Hospital, College of Medicine, Zhejiang University, Hangzhou, 310058 Zhejiang China; 39 The First Affiliated Hospital of Anhui Medical University, Hefei, 230032 Anhui China; 40 Department of Psychosomatics and Psychiatry, Zhongda Hospital, School of Medicine, Southeast University, Nanjing, 210009 Jiangsu China; 41 First Hospital of Shanxi Medical University, Taiyuan, 030001 Shanxi China; 42 West China Hospital of Sichuan University, Chengdu, 610044 Sichuan China; 43 Department of Psychiatry, Shanghai Jiao Tong University School of Medicine, Shanghai, 200025 China; 44 Developmental Population Neuroscience Research Center, IDG/McGovern Institute for Brain Research, Beijing Normal University, 100091 Beijing, China; 45 National Basic Science Data Center, 100038 Beijing, China; 46 Center for Cognition and Brain Disorders, The Affiliated Hospital of Hangzhou Normal University, Hangzhou, 310018 Zhejiang China; 47 Zhejiang Key Laboratory for Research in Assessment of Cognitive Impairments, Hangzhou, 310000 Zhejiang China

**Keywords:** Depression, Diagnostic markers

## Abstract

Major depressive disorder (MDD) is a globally prevalent and highly disabling disease characterized by dysfunction of large-scale brain networks. Previous studies have found that static functional connectivity is not sufficient to reflect the complicated and time-varying properties of the brain. The underlying dynamic interactions between brain functional networks of MDD remain largely unknown, and it is also unclear whether neuroimaging-based dynamic properties are sufficiently robust to discriminate individuals with MDD from healthy controls since the diagnosis of MDD mainly depends on symptom-based criteria evaluated by clinical observation. Resting-state functional magnetic resonance imaging (fMRI) data of 221 MDD patients and 215 healthy controls were shared by REST-meta-MDD consortium. We investigated the spatial-temporal dynamics of MDD using co-activation pattern analysis and made individual diagnoses using support vector machine (SVM). We found that MDD patients exhibited aberrant dynamic properties (such as dwell time, occurrence rate, transition probability, and entropy of Markov trajectories) in some transient networks including subcortical network (SCN), activated default mode network (DMN), de-activated SCN-cerebellum network, a joint network, activated attention network (ATN), and de-activated DMN-ATN, where some dynamic properties were indicative of depressive symptoms. The trajectories of other networks to deactivated DMN-ATN were more accessible in MDD patients. Subgroup analyses also showed subtle dynamic changes in first-episode drug-naïve (FEDN) MDD patients. Finally, SVM achieved preferable accuracies of 84.69%, 76.77%, and 88.10% in discriminating patients with MDD, FEDN MDD, and recurrent MDD from healthy controls with their dynamic metrics. Our findings reveal that MDD is characterized by aberrant dynamic fluctuations of brain network and the feasibility of discriminating MDD patients using dynamic properties, which provide novel insights into the neural mechanism of MDD.

## Introduction

Major depressive disorder (MDD) is one of the most common mental illnesses worldwide, the leading cause of disability, and the single largest contributor to global disease burden in the context of increasing global turmoil [1]. Although significant progress has been made in the study of the neural mechanism of depression and the development of anti-depressants in the past decades, the understanding of the pathophysiology underlying depression remains elusive. Non-invasive measurement of neuronal activity with functional magnetic resonance imaging (fMRI) has emerged as a useful modality for investigating psychiatric disorders such as MDD [2–4]. Numerous resting-state fMRI studies have provided rich evidence of aberrant functional connectivity (FC) or abnormal network communication in patients with MDD, especially within default mode network (DMN) [5–8]. Task-based fMRI experiments also show abnormalities of brain activity patterns in MDD patients during facial recognition [9], cognitive control [10], and reward processing [11]. Moreover, aberrant brain functional features can be used as reliable neurobiological markers for the diagnosis of MDD [12–14] and help to determine potential therapeutic targets for treatment [15, 16]. All these fMRI studies assumed that the interactions between the brain regions are temporally stationary throughout the entire scan [3, 4].

However, the intrinsic brain activity is highly non-stationary, which leads to dynamic alterations in signals measured by fMRI [17]. The investigation of dynamic FC has the potential to provide novel insight into how brain networks coalesce and dissolve over time. Dynamic FC has been widely explored in patients with MDD. A large number of studies on dynamic FC adopted a sliding-window approach [18] and found that MDD was characterized by abnormal brain fluctuations in DMN and time-varying brain activities were significantly associated with depression severity [19–21]. The sliding-window approach exploited the first-order (pairwise) relationships between fMRI time series in a consecutive small window, wherein its results depend on the window shape and size. In addition, this approach has lower test-retest reliability [22], and it cannot detect abruptions in two adjacent windows or within a consecutive window. To address the above limitations, numerous methods have been developed, including dynamic conditional correlation (DCC) approach [23] and co-activation pattern (CAP) analysis [24]. DCC utilizes multivariate volatility method (GARCH: a generalized auto-regressive conditional heteroscedastic model) which can resolve brain dynamics at a single time point and all the parameters in DCC are estimated by quasi-maximum likelihood. However, fitting of GARCH model makes whole-brain computation of DCC much more time-consuming without dimensionality reduction and parameter estimation has been shown to have severe negative biases [25]. Different from model-based DCC analysis, CAP uses k-means clustering, and thus it is data-driven, relies on few model assumptions, and is time efficient. This approach can flexibly capture spontaneous spatiotemporal dynamics of brain activity at frame resolution (single fMRI volume) with good sensitivity and specificity, and it might provide fine-grained information on brain dynamics.

The CAP method has been applied to investigate the brain dynamics in MDD patients [26–30]. All these studies documented that MDD was characterized by abnormal dynamic fluctuations within DMN and between DMN and other networks, and specific dynamic properties were correlated with depressive symptomatology. However, the dynamic properties in DMN were inconsistent in these single-site studies. Specifically, Zheng et al. documented a reduced persistence of DMN [28], while other studies reported increased dwell time, frequency, or persistence of DMN [26, 27, 29, 30] in MDD patients. Such inconsistency may be partly due to sample heterogeneity (such as age, gender, medication, episode, age of onset, etc.), data collected from single site, data preprocessing method, and limited statistical power with small sample size [31].

In the current study, we employed the CAP method to investigate the dynamic fluctuations of brain functional activity in a large MDD cohort (*n* = 436) from multiple centers and evaluated the feasibility of using these dynamics for MDD diagnosis. Subgroup analyses were also conducted for patients with recurrent MDD and first-episode drug-naïve (FEDN) MDD. Finally, we evaluated the robustness of our results under different parcellation schemes and cluster numbers.

## Material and methods

### Data resource

The data were obtained from the REST-meta-MDD Project [8] of the DIRECT Consortium [32]. A total of 1300 MDD patients and 1128 healthy controls from 24 sites in China were recruited in the project, and R-fMRI indices were shared through the R-fMRI Maps Project (http://rfmri.org/REST-meta-MDD).

### Participants

All patients with MDD were hospital-diagnosed according to the Structured Clinical Interview of Diagnostic with Statistical Manual of Mental Disorder (DSM)-IV or International Classification of Disease 10.

We restricted our analyses to those subjects with 240 resting-state fMRI time points and a repetition time of 2 s to reduce biases caused by different time points or temporal resolutions when constructing dynamic analysis. Ultimately, 436 subjects consisting of 215 healthy controls and 221 MDD patients from 6 sites were included in this study based on our exclusion criteria (see supplementary material). Here, MDD patients were further divided into subgroups of 43 first-episode drug-naïve (FEDN) MDD patients and 100 recurrent MDD patients from 4 sites.

The demographic information included gender, age, education, and site in both MDD patients and healthy controls. Depression severity was assessed using the 17-item Hamilton Depression Rating Scale (HAMD) in MDD group.

### Image acquisition and processing

Resting-state fMRI data were acquired at each site using different scanners and parameters. Pre-processing step was described in supplementary step [8]. Details on the sites and imaging parameters can be found in Supplementary Table S1.

In the post-processing step, fMRI signals were extracted from 116 regions of interest (ROIs) using automatic anatomic labeling (AAL) atlas [33]. These 116 ROIs were allocated to 6 networks including ATN, DMN, sensorimotor network (SMN), visual network (VN), subcortical network (SCN), and cerebellum network (CN).

### Co-activation pattern (CAP) analysis

We adopted an ROI-wise CAP analysis due to data limitations (raw data was not shared to protect participant privacy) [24]. First, time series extracted from 116 ROIs were converted to z-score (zero mean and unit standard deviation) and then temporally clustered using k-means clustering (one minus the Pearson correlation coefficient between frames as clustering distance metric, 500 iterations, and 5 repetitions with random initializations). The *Z* statistic CAP maps for individual clusters were generated by averaging the spatial maps of all subjects and then normalized using the standard error.

The choice of the number of clusters *k* is a core problem in clustering. We initially evaluated the clustering performance using elbow criteria, Silhouette score, Calinski–Harabasz, Davies–Bouldin, and Dunn Validity index for different *k* values ranging from 2 to 20. As shown in Supplementary Fig. S1A, it was difficult to determine an optimal *k* using the above evaluation metrics. As such, a trade-off value of *k* = 7 was used in this study, considering that *k* generally ranged from 6 to 8 in previous studies [34–36].

To provide a rich characterization of temporal properties of CAP, we quantified MDD dynamics using dwell time, occurrence rate, transition probability matrix, and entropy of Markov trajectories. Calculations on these metrics were described in supplementary material. The CAP analysis was processed using custom-written scripts in MATLAB R2016a (The MathWorks, Inc., Natick, MA, USA).

### Support vector machine (SVM)

A Gaussian radial basis function-based SVM classifier was implemented for classification procedure using the python package scikit-learn, because of its promising results in neuroimaging [37, 38]. Here, we applied the nested cross-validation (CV) strategy to conduct models. Features were independently standardized to a normal distribution by z-score normalization, whose parameters were learned from the training set and then applied to the testing set. Outer loop of nested CV was performed to split training and test sets, and quantify classification performance. The classification performance was described by area under curve (AUC), accuracy (Acc), sensitivity (Sen), harmonic mean of precision and recall (F1 score), positive predictive value (PPV), and negative predictive value (NPV). Hyper-parameters (optimal cost parameter, *C*; tolerance, gamma) were optimized using a grid search algorithm based on the highest accuracy in the training set of inner loops.

### Reproducibility analysis

We implemented several strategies to test the robustness of our results. 1) Different cluster numbers. We examined whether cluster numbers (*k* = 6 and *k* = 8) may affect the overall trend of dynamic properties in MDD. 2) Different parcellation schemes. We constructed brain dynamics using a structure-based atlas (the Harvard-Oxford structural parcellation [39]) and a functional atlas (the Craddock functional parcellation [40]). 3) Global signal regression (GSR) in the preprocessing step. The reproducibility of dynamic metrics such as dwell time, occurrence rate, persistence probability, and entropy of Markov trajectories were assessed in the above three scenarios.

### Statistical analysis

To examine the statistical difference in dynamic CAP properties between MDD patients and healthy controls, two-tailed independent sample t-tests were employed after controlling for age, gender, education level, site, and frame-wise displacement. T-tests met assumptions for normality and equal variances. Pearson correlation was used to determine whether these dynamic properties were correlated with the clinical scores of HAMD in MDD patients after adjusting for age, gender, education level, site, and frame-wise displacement. Additionally, spatial similarity between CAPs was assessed using Pearson correlation. All the results within a specific dynamic property were corrected for multiple comparisons using false discovery rate (FDR) [41]. Statistical significance was considered at *P* < 0.05 (two-tailed). These statistical analyses were carried out in MATLAB R2016a (The MathWorks, Inc., Natick, MA, USA) and Social Sciences, version 26.0 (SPSS, Chicago, IL).

### Ethics

We used publicly-available data from Rest-meta-MDD project of DIRECT Consortium (Approval ID: U0240). All the sites agreed to share final R-fMRI indices from studies approved by local Institutional Review Boards.

## Results

### Demographics and clinical characteristics

The demographic and clinical characteristics of MDD patients and healthy controls are summarized in Table 1. No group difference was reported in gender (${\chi }^{2}$ = 2.267, *P* = 0.143) or age (*Z* = −0.011, *P* = 0.991) between MDD patients and healthy controls. MDD patients had lower education levels than healthy controls (*Z* = −3.125, *P* = 0.002). The mean HAMD-17 of MDD patients was 21.71 ± 6.60.

**Table 1:** Demographic and clinical description of healthy controls and MDD patients.

Characteristic	Healthy controls (*N* = 215)	MDD patients (*N* = 221)	*P* value
Gender (male/female)	93/122	80/141	0.143^a^
Age (years)	33.48 ± 12.88	33.45 ± 12.78	0.991^b^
Education level (years)	12.87 ± 3.29	11.97 ± 3.07	0.002^b^
HAMD-17	NA	21.71 ± 6.60	NA

Data were represented as mean ± SD (standard deviation).

*MDD* major depressive disorder, *HAMD* Hamilton Depression Scale.

^a^The *p*-value of gender was obtained by the Pearson chi-square cross-table test.

^b^The *p*-values of age and education level were obtained by the Mann–Whitney U test.

### Seven dynamically recurring functional brain states

**Fig. 1:**
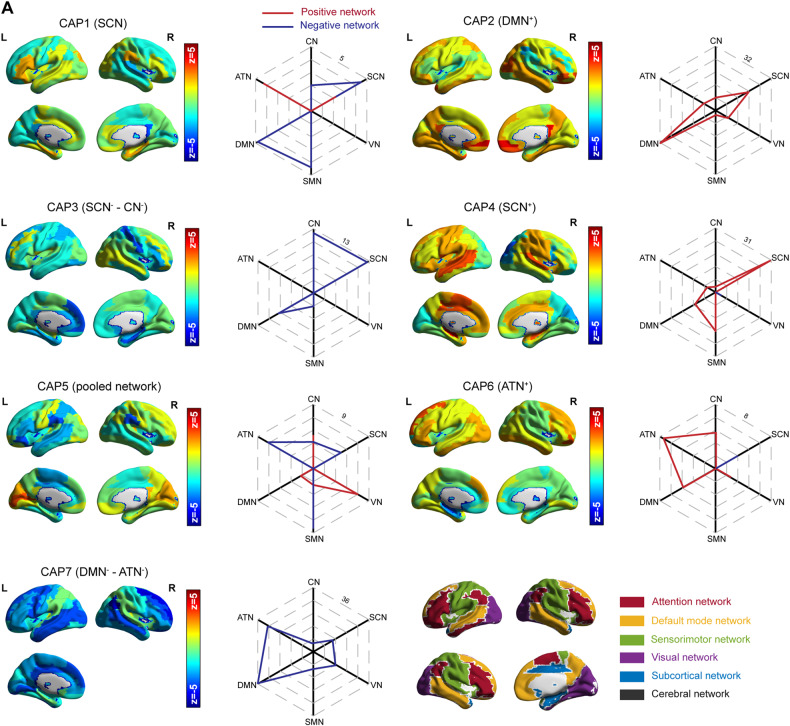
The seven identified CAP topographies. **A** Main network components of each CAP. The CAPs consisted of CAP1 (SCN), CAP2 (DMN^+^), CAP3 (SCN^−^-CN^−^), CAP4 (SCN^+^), CAP5 (pooled network), CAP6 (ATN^+^) and CAP7 (DMN^−^-ATN^−^). SCN subcortical network, DMN default mode network, CN cerebellum network, SMN somatosensory network, ATN attention network.

To quantify time-varying heterogeneous information in resting state fMRI data, we applied CAP analysis to divide the brain into multiple overlapping states. Using k-means clustering, seven recurring states were identified (Fig. 1A). We adopted two methods to determine which network the CAP belongs to. 1) The proportion of summed–absolute Z statistic (*Z* > 1.96) of ROIs inside each network to summed–absolute Z statistic (*Z* > 1.96) of all ROIs. 2) The activation matrix (7_*k*_ × 116_ROI_) with absolute *Z* statistic (*Z* > 1.96) of each ROI multiplied by the network mask (116_ROI_ × 6_networks_), positive network, and negative network were calculated separately.

CAP1 was mainly dominated by SCN (SCN; both activated and de-activated, proportion = 30.02%). CAP2 was characterized by positive activation of DMN (hereafter activation of a network was expressed with ‘network^+^’, de-activation was expressed with ‘network^−^’; DMN^+^; proportion = 43.36%). CAP3 was the de-activation of SCN (proportion = 35.25%) and CN (proportion = 34.20%) (SCN^−^-CN^−^). CAP4 was mainly overlapped with activated SCN (SCN^+^; proportion = 42.93%). CAP5 was a joint network containing CN, ATN, SMN, and VN (pooled network; proportions = 20.85%, 18.51%, 26.48%, and 17.91, respectively). CAP6 was mainly dominated by activated ATN (ATN^+^; proportion = 34.55%). CAP7 mainly corresponded with de-activated DMN and ATN (DMN^−^-ATN^−^; proportion = 32.95% and 26.91%, respectively) (Supplementary Fig. S2A). The obtained CAPs had some pairs of anti-correlated spatial configurations. The first CAP pair showed anti-correlation between CAP2 (DMN^+^) and CAP7 (DMN^−^-ATN^−^) (*r* = −0.5230, *P* < 0.0001). The second CAP pair showed anti-correlated configuration between CAP4 (SCN^+^) and CAP5 (pooled network) (*r* = −0.5153, *P* < 0.0001) (Supplementary Table S2).

### Dwell time and occurrence rate of each CAP state

**Fig. 2:**
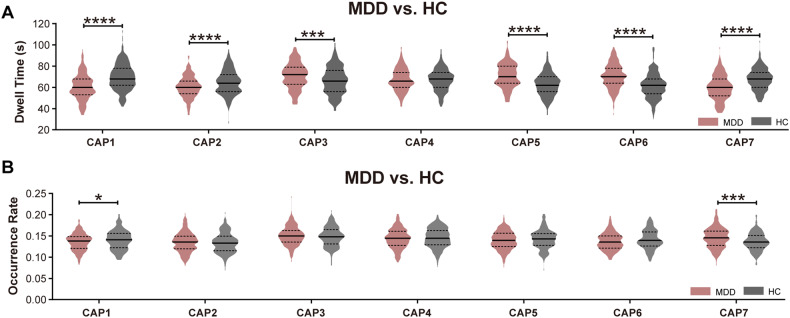
Dwell time and occurrence rate of CAPs. **A** Group difference in dwell time between MDD patients and healthy controls for seven CAPs. **B** Group difference in occurrence rate between MDD patients and healthy controls in each CAP. Two sample t tests with covariates were used for dwell time and occurrence rate comparisons. All results were corrected for multiple comparisons using FDR correction within seven CAPs. Data were represented as mean ± SEM (standard error of mean). *P < 0.05, **P < 0.01, ***P < 0.001, ****P < 0.0001. n_MDD_ = 221; n_healthy control_ = 215. See Supplementary Table S3 and 4 for detailed statistics. MDD major depressive disorder; HC healthy controls. CAP1: SCN; CAP2: DMN^+^; CAP3: SCN^−^-CN^−^; CAP4: SCN^+^; CAP5: pooled network; CAP6: ATN^+^; CAP7: DMN^−^-ATN^−^.

We first tested whether dynamic properties such as dwell time and occurrence rate were different between MDD patients and healthy controls. Overall, MDD patients showed a significantly reduced dwell time in CAP1 (SCN), CAP2 (DMN^+^), and CAP7 (DMN^−^-ATN^−^), and a significantly increased dwell time in CAP3 (SCN^−^-CN^−^), CAP5 (pooled network) and CAP6 (ATN^+^) (Fig. 2A). Relative to healthy controls, MDD patients had a decreased occurrence rate in CAP1 (SCN) and increased occurrence rate in CAP7 (DMN^-^-ATN^-^) (Fig. 2C, Supplementary Table S3).

The behavior relevance with dwell time was evaluated in MDD patients using Pearson correlation after adjusting for age, gender, education level, site, and frame-wise displacement. As shown in Supplementary Table S4, dwell time of CAP3 (SCN^−^-CN^−^) (*r* = 0.1715, *P* = 0.0107) yields strong positive relationship with depression severity, while dwell time of CAP4 (SCN^+^) (*r* = −0.1882, *P* = 0.0050) is negatively correlated with depression severity.

We also assessed the difference in dwell time and occurrence rate between MDD patients and healthy controls at site level (Supplementary Fig. S4A). The decreased dwell time of CAP1 (SCN) in MDD patients was consistently observed in 5 of the 6 sites except site 1 due to data imbalance (n_MDD_ = 31, n_healthy controls_ = 6), followed by CAP5 (pooled network) and CAP6 (ATN^+^) (4 of the 6 sites), CAP7 (DMN^−^-ATN^−^) and CAP3 (SCN^−^-CN^−^) (3 of the 6 sites). The dwell time of CAP7 (DMN^−^-ATN^−^) of healthy controls showed an increasing trend among all sites, albeit less significant at some sites. The results of occurrence rate were less repeatable across all sites.

### Transition probabilities and entropy of Markov trajectories between each CAP state

**Fig. 3:**
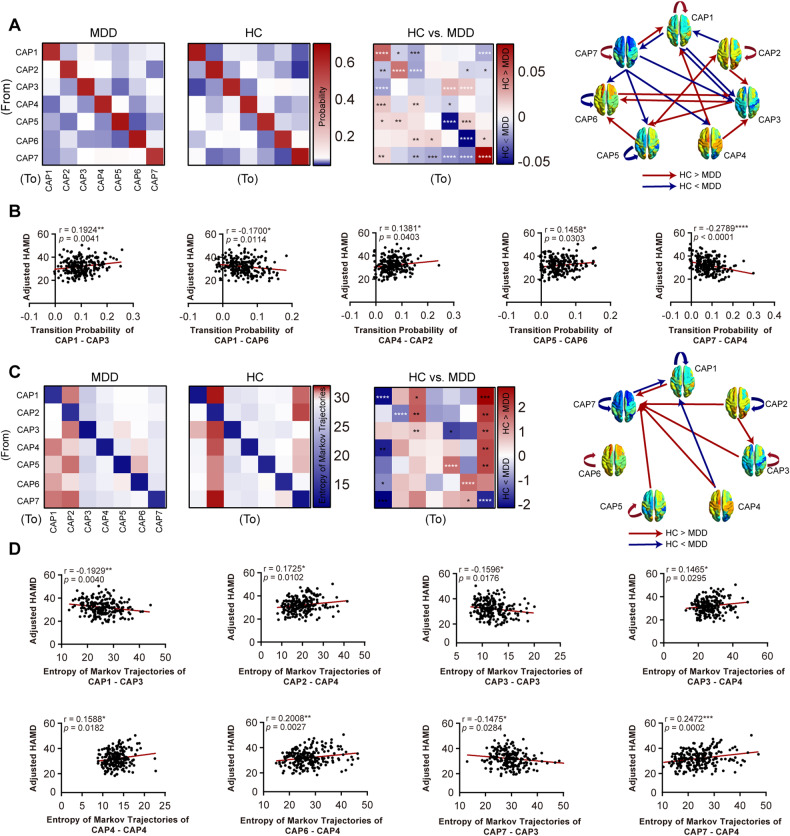
Transition probabilities and corresponding temporal trajectories among CAPs. **A** Transition probability matrix of MDD patients and healthy controls between each CAP and comparison between two groups. The diagonal entries are persistence probabilities. Red rectangle indicates that the transition probability from a CAP to another CAP in healthy controls was greater than MDD patients. The blue rectangle is the opposite. **B** Behavioral evidence of transition probabilities for MDD patients. **C** Entropy of Markov trajectories matrix and comparison between MDD patients and healthy controls. **D** Behavioral evidence of temporal trajectories for MDD patients. Two sample t tests with covariates were used for comparison of transition probabilities and temporal trajectories. The relationship between these properties and HAMD scores was assessed using Pearson correlation. All *P* values were corrected for multiple comparisons with FDR correction within 49 paths. *P < 0.05, **P < 0.01, ***P < 0.001, ****P < 0.0001. n_MDD_ = 221; n_healthy control_ = 215. CAP1: SCN; CAP2: DMN^+^; CAP3: SCN^−^-CN^−^; CAP4: SCN^+^; CAP5: pooled network; CAP6: ATN^+^; CAP7: DMN^−^-ATN^−^. See Supplementary Table S5 and 6 for detailed statistics.

MDD patients had lower persistence probabilities within CAP1 (SCN), CAP2 (DMN^+^), CAP7 (DMN^−^-ATN^−^) and higher persistence probabilities within CAP5 (pooled network) and CAP6 (ATN^+^) (Fig. 3A). Note that the transition probability was asymmetric, which means that the transition was directional. MDD patients showed significantly increased transition probabilities from 1) CAP1 to CAP2, CAP3 and CAP7, 2) CAP2 to CAP1, CAP3, CAP6 and CAP7, 3) CAP3 to CAP1, 4) CAP7 to CAP3, CAP4, CAP5 and CAP6. On the other hand, MDD patients showed weaker transition probabilities from 1) CAP3 to CAP5 and CAP6, 2) CAP4 to CAP1 and CAP3, 3) CAP5 to CAP1, CAP2 and CAP6, 4) CAP6 to CAP3, CAP4 and CAP7, and 5) CAP7 to CAP1 (CAP1: SCN; CAP2: DMN^+^; CAP3: SCN^−^-CN^−^; CAP4: SCN^+^; CAP5: pooled network; CAP6: ATN^+^; CAP7: DMN^−^-ATN^−^) (Fig. 3A). The detailed statistics are described in Supplementary Table S5. As shown in Fig. 3B, Pearson correlation reveals a significant correlation between depression severity and transition probabilities from CAP1 (SCN) to CAP3 (SCN^−^-CN^−^) (*r* = 0.1924, *P* = 0.0041), CAP1 (SCN) to CAP6 (ATN^+^) (*r* = −0.17, *P* = 0.0114), CAP4 (SCN^+^) to CAP2 (DMN^+^) (*r* = 0.1381, *P* = 0.0403), CAP5 (pooled network) to CAP6 (ATN^+^) (*r* = 0.1458, *P* = 0.0303), CAP7 (DMN^−^-ATN^−^) to CAP4 (SCN^+^) (*r* = −0.2789, *P* < 0.0001).

To describe the variation in transitions, we further characterized these transition matrices using entropy of Markov trajectories. Surprisingly, we found that the entropies of the remaining CAPs to CAP7 (DMN^−^-ATN^−^) were significantly (or trend-level) higher in healthy controls, indicating that the trajectories of the remaining CAPs to CAP7 (DMN^−^-ATN^−^) were more accessible in MDD patients (Fig. 3C). Decreased accessibilities were found within CAP1 (SCN), CAP2 (DMN^+^) and CAP7 (DMN^−^-ATN^−^) in MDD patients, while CAP3 (SCN^−^-CN^−^), CAP5 (pooled network) and CAP6 (ATN^+^) exhibited increased accessibility in MDD patients **(**Fig. 3C). Decreased entropy of Markov trajectories from CAP1 (SCN) to CAP3 (SCN^−^-CN^−^) (*r* = −0.1929, *P* = 0.004), within CAP3 (SCN^−^-CN^−^) (*r* = −0.1596 *P* = 0.0176), CAP7 (DMN^−^-ATN^−^) to CAP3 (SCN^−^-CN^−^) (*r* = −0.1475, *P* = 0.0284) were associated with elevated depression severity. Enhanced entropy of Markov trajectories from CAP2 (DMN^+^) to CAP4 (SCN^+^) (*r* = 0.1725, *P* = 0.0102), CAP3 (SCN^−^-CN^−^) to CAP4 (SCN^+^) (*r* = 0.1465, *P* = 0.0295), within CAP4 (SCN^+^) (*r* = 0.1588, *P* = 0.0182), CAP6 (ATN^+^) to CAP4 (SCN^+^) (*r* = 0.2008, *P* = 0.0027) and CAP7 (DMN^−^-ATN^−^) to CAP4 (SCN^+^) (*r* = 0.2472, *P* = 0.0002) were correlated with increased depression severity (Fig. 3D).

We repeated transition probability analysis among all sites (Supplementary Fig. S5A). The persistence probabilities of CAP2 (DMN^+^), CAP5 (pooled network), CAP6 (ATN^+^) and CAP7 (DMN^−^-ATN^−^) have a good reproducibility in MDD patients (4 of the 6 sites). Consistent changes in transition probability were found from 1) CAP2 (DMN^+^) to CAP7 (DMN^−^-ATN^−^), 2) CAP3 (SCN^−^-CN^−^) to CAP5 (pooled network) and CAP6 (ATN^+^), and 3) CAP7 (DMN^−^-ATN^−^) to CAP1 (SCN) and CAP6 (ATN^+^) (3 of the 6 sites). Results on entropy of Markov trajectories were validated in each site (Supplementary Fig. S5B). Similar trends of higher accessibility in MDD patients between the rest CAPs to CAP7 (DMN^−^-ATN^−^) were found in site 1, site 2, site 3, and site 5.

### Subgroup analyses

#### Subgroup analyses of dwell time and occurrence rate

**Fig. 4:**
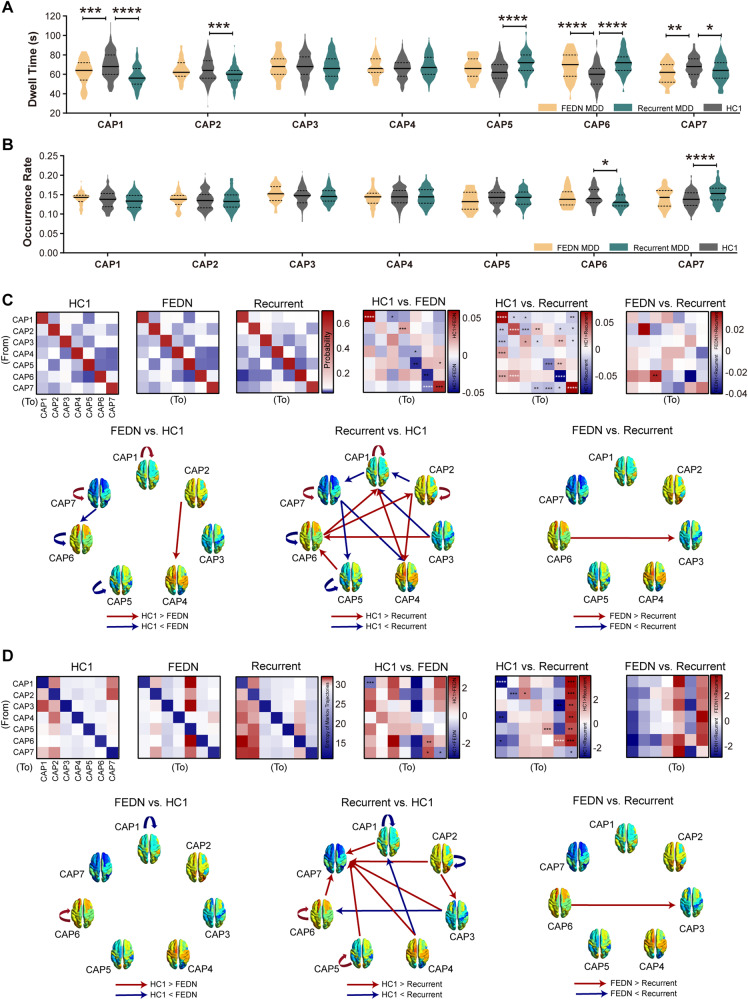
Subgroup differences (FEDN MDD vs. Recurrent MDD vs. HC1) in temporal characteristics. **A** Dwell time. **B** Occurrence rate. **C** Transition probabilities and their behavioral relevance. **D** Entropy of Markov trajectories (accessibility). All results were corrected for multiple comparisons using FDR correction. Data were represented as mean ± SEM. *P < 0.05, **P < 0.01, ***P < 0.001, ****P < 0.0001. n_FEDN_ = 43; n_HC1_ = 135; n_Recurrent_ = 100. HC1: healthy controls from the same site of FEDN and recurrent MDD patients. CAP1: SCN; CAP2: DMN^+^; CAP3: SCN^−^-CN^−^; CAP4: SCN^+^; CAP5: pooled network; CAP6: ATN^+^; CAP7: DMN^−^-ATN^−^.

We also compared the dwell time between FEDN MDD patients, recurrent MDD patients, and healthy controls. The 135 healthy controls (HC1) were selected from sites that contained patients with FEDN or recurrent MDD patients. FEDN patients showed significantly lower total dwell time in CAP1 (SCN) and CAP7 (DMN^−^-ATN^−^), and higher total dwell time in CAP6 (ATN^+^) (Fig. 4A). The results on dwell time of recurrent MDD patients were consistent with those of all pooled MDD patients, except that there was no significant difference in CAP3 (SCN^−^-CN^−^). In CAP6 (ATN^+^), the occurrence rate was significantly weaker in recurrent MDD patients, higher in CAP7 (DMN^−^-ATN^−^) (Fig. 4A, B). There were no significant changes in occurrence rate in FEDN MDD patients (Fig. 4B).

#### Subgroup analyses of persistence probabilities and transition probabilities

Results of persistence probabilities in FEDN MDD patients were similar to those in all pooled MDD patients, except no difference was found in CAP2 (DMN^+^) (Fig. 4C). The same results of persistence probabilities between MDD and healthy controls were available for recurrent MDD. In addition, the persistence probability of CAP3 (SCN^−^-CN^−^) was lower in recurrent MDD (Fig. 4C).

In patients with FEDN MDD, we found increased transition probabilities from CAP1 (SCN) to CAP3 (SCN^−^-CN^−^), CAP4 (SCN^+^) to CAP5 (pooled network), CAP7 (DMN^−^-ATN^−^) to CAP6 (ATN^+^), decreased transition probabilities from CAP2 (DMN^+^) to CAP4 (SCN^+^), and CAP5 (pooled network) to CAP7 (DMN^−^-ATN^−^) (Fig. 4C). Recurrent MDD patients showed similar results to MDD patients in transition probabilities. We also found a lower transition probability from CAP6 (ATN^+^) to CAP3 (SCN^−^-CN^−^) in recurrent MDD patients than in FEDN MDD patients. The detailed statistics are described in Supplementary Tables S3–5. Behavior relevance is described in Supplementary Fig. S7.

#### Subgroup analyses of entropy of Markov trajectories

Compared with healthy controls, the accessibilities within CAP1 (SCN) and CAP7 (DMN^−^-ATN^−^) were lower in FEDN MDD patients. Increased accessibilities within CAP6 (ATN^+^), between CAP7 (DMN^−^-ATN^−^) and CAP6 (ATN^+^) were found in FEDN MDD patients. Consistent results with MDD patients in entropy of Markov trajectories were found in recurrent MDD patients, except no difference was found within CAP3 (SCN^−^-CN^−^). There was no difference in entropy of Markov trajectories between FEDN and recurrent MDD patients (Fig. 4D, Supplementary Table S6).

### Reproducibility evaluation

**Fig. 5:**
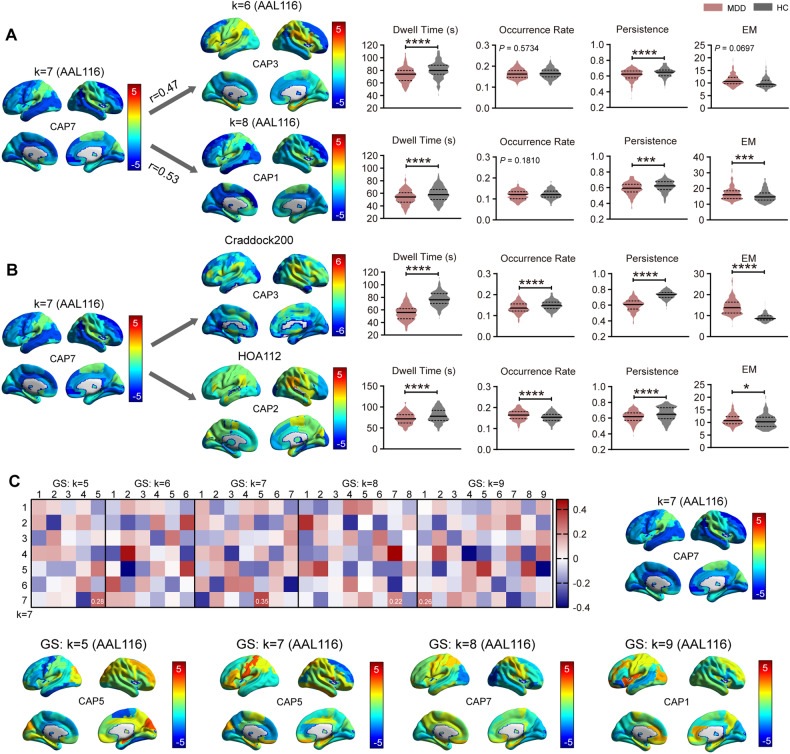
Reproducibility analysis. **A** Different cluster numbers *k*. **B** Different parcellation schemes. Craddock’s 200 functional clustering atlas and Harvard-Oxford atlas were tested. **C** Global signal regression. Two sample t test with covariates. Multiple comparisons with FDR correction. Data were represented as mean ± SEM. *P < 0.05, **P < 0.01, ***P < 0.001, ****P < 0.0001. n_MDD_ = 221; n_healthy control_ = 215. EM entropy of Markov trajectories. CAP1: SCN; CAP2: DMN^+^; CAP3: SCN^−^-CN^−^; CAP4: SCN^+^; CAP5: pooled network; CAP6: ATN^+^; CAP7: DMN^−^-ATN^−^.

We first examined dynamic characteristics with different values of cluster number *k*. The CAP7 (DMN^−^-ATN^−^) was chosen for reproducibility assessment. Spatially similar CAPs with CAP7 (DMN^−^-ATN^−^) were identified when *k* = 6 and *k* = 8 (Fig. 5A). We found a trend consistent with the previous results in CAP7 (DMN^−^-ATN^−^) (Figs. 2 and 3), except inconsistent results in occurrence rate (Fig. 5A). All spatial patterns of CAPs when *k* = 6 and *k* = 8 are displayed in Supplementary Fig. S8A and their temporal dynamics were overall consistent with those of *k* = 7 (Supplementary Fig. S8B–C).

Two spatially similar maps can be observed using Craddock200 atlas and HOA112 atlas (Fig. 5B). The results of dwell time, persistence probability, and entropy of Markov trajectories between MDD patients and healthy controls using these two parcellations were shown to be qualitatively consistent with the above findings (Figs. 2 and 3) while occurrence rate of Craddock200 atlas yielded contrary results.

GSR was also considered in our analysis, but no significant difference was found in the dynamic properties between the two groups. One possible reason is that the centroids were changed after clustering, and another is that there are too few ROIs to perform Pearson correlation, thus it cannot identify a similar spatial map very well. We noted that among the several spatial maps with high Pearson correlation values, unwanted activations in some ROIs were exhibited compared with spatial map of CAP7 (DMN^-^-ATN^-^) when *k* = 7 (Fig. 5C). Moreover, although GSR can effectively remove global artifacts arising from motion and respiration [42, 43], it is still considered to be a controversial step in fMRI analysis since the global signal may contain important neurobiological information in MDD [44].

### Binary MDD classification of four groups using dynamic measures

**Fig. 6:**
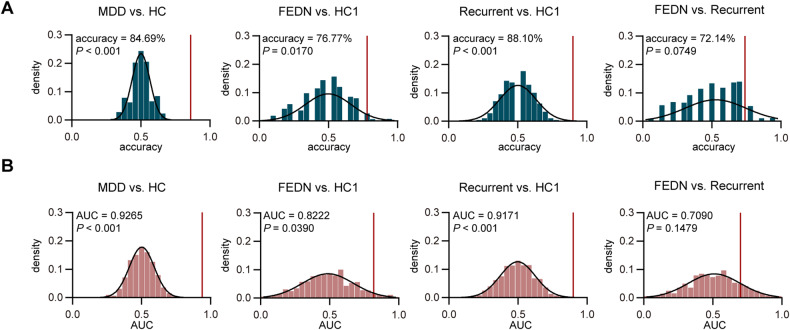
Accuracy and AUC based on dynamic features using SVM. **A** Accuracy distribution with 1000 permutation tests of four classifiers (MDD vs. HC, FEDN vs. HC1, Recurrent vs. HC1 and FEDN vs. Recurrent). **B** AUC distribution with 1000 permutation tests of four classifiers. *P* values were based on 1000 permutation tests.

We inputted all pooled dynamic properties including dwell time, occurrence rate, persistence probabilities, and entropy of Markov trajectories with *P* values less than 0.05 into SVM classifier and obtained mean accuracies of 84.69%, 76.77%, 88.10%, and 72.14% in distinguishing MDD patients from healthy controls, FEDN MDD patients from healthy controls, recurrent MDD patients from healthy controls, and FEDN from recurrent MDD patients, respectively (Fig. 6A). The mean AUCs were 0.93, 0.82, 0.92 and 0.71 for these four groups, respectively (Fig. 6B). Sensitivity, specificity, F1 score, PPV and NPV are displayed in Supplementary Tables S7–10.

## Discussion

In this study, we employed a large multicenter cohort (*n* = 436) to investigate the dynamic properties of brain functional activity in MDD patients using a data-driven CAP method. In current study, we identified seven CAPs encompassing CAP1 (SCN), CAP2 (DMN^+^), CAP3 (SCN^−^-CN^−^), CAP4 (SCN^+^), CAP5 (pooled network), CAP6 (ATN^+^), and CAP7 (DMN^−^-ATN^−^) in the MDD cohort. MDD patients showed aberrant dynamic properties in CAPs including CAP1 (SCN), CAP2 (DMN^+^), CAP3 (SCN^−^-CN^−^), CAP5 (pooled network), CAP6 (ATN^+^), and CAP7 (DMN^−^-ATN^−^). Some dynamic characteristics in specific CAPs were associated with depressive symptoms. Interestingly, CAP7 (DMN^−^-ATN^−^) was the most probable destination of the other six CAPs in both MDD and recurrent MDD patients. These results suggest that MDD is characterized by abnormal timing-varying intrinsic fluctuations of large-scale brain functional networks. We also found that the time-varying properties could distinguish MDD patients from healthy controls with an accuracy of 84.69%, implying that these time-varying properties might be used as reliable biomarkers of MDD.

### Significant alterations of brain dynamics in MDD population

CAP analysis can reflect spatial-temporal heterogeneous information in fMRI data [24]. These CAPs represent flexibility and dynamic coordination between distinct neural systems [45].

DMN is involved in introspection, self-focused thoughts, emotional control, and motivation. It has long been reported to contribute to the development of depression [5–8, 26, 46]. Compared with healthy controls, MDD patients were found to have shorter dwell time and fewer transitions in activated DMN and de-activated DMN-ATN. In addition, de-activated DMN-ATN occurred more frequently and transitioned more from one of these two CAPs to another CAP in MDD patients than healthy controls. These results suggested that the co-activation or co-deactivation of DMN is temporally unstable in MDD patients, which is in line with several previous studies [19, 20, 28, 47]. Another finding of our study is that de-activated DMN-ATN, as an attractor, is more accessible in patients with MDD and recurrent MDD than healthy controls, and a similar trend was observed between patients with FEDN MDD and recurrent MDD. To the best of our knowledge, no studies have applied the entropy of Markov trajectories in the CAP method to study MDD. Thus, this finding is novel compared to previous results and further confirms that highly variable DMN fluctuations may exaggerate internal self-referential thoughts that may trigger MDD.

Several studies investigated the dynamics of MDD using the CAP approach. Kaiser et al. documented that MDD patients showed increased dwell time and persistence in frontoinsular-DMN, and higher transition frequency between frontoinsular-DMN and prototypical DMN was associated with elevated depressive symptoms [26]. Zheng et al. found reduced occurrence rate and transitions within DMN [28], while Emily L. Belleau reported that MDD patients dwelled more time in DMN-FPN and had a higher transition probability between DMN-FPN to prototypical DMN [27]. Goodman et al. found that elevated depressive symptoms were positively correlated with enhanced frequency and dwell time of the DMN [29]. Hou et al. found that dominant CAP in the left posterior DMN was positively correlated with depressive symptoms [30]. Consistent with part of findings of Zheng et al. (reduced transitions within DMN), we also found a reduced persistence probability of both activated and de-activated DMN in patients with MDD [28]. Our results are inconsistent with the results of the other four studies [26, 27]. The possible reasons are as follows: 1) most of data were collected from single site with limited reliability, 2) different MDD risk genes in Han Chinese compared with Caucasian subjects [48], 3) somatic symptoms in Han Chinese rather than psychological symptoms [49], 4) heterogeneity in clinical characteristics.

We also found that MDD patients had shorter dwell time in SCN and longer dwell time in de-activated SCN-CN, the joint network, and activated ATN, suggesting dynamic network disruption in patients with MDD. Besides, we observed transition trajectories between CAPs were dynamically and nonmonotonically changed in MDD patients. These abnormal network communications (transitions) have been reported to be engaged in MDD in previous static and dynamic fMRI studies [5, 8, 50–52]. Attention network is involved in working memory and cue attention, and it also intrinsically affects MDD [53, 54]. In addition, the crucial role of subcortical network and somatosensory network in processing and regulating emotions has also been recognized [55–57]. Cerebellum, which also involves mood regulation and cognitive processing, has been proven to be associated with depression [58, 59]. All these evidences suggest that dysfunction of these networks may contribute to mood regulation and motivation functions of MDD. Our results showed that dwell time of SCN and deactivated SCN-CN were positively related to depression severity, whereas dwell time of ATN and de-activated DMN-ATN were almost negatively associated with depression severity. Besides, persistence probabilities within SCN and activated ATN were associated with depressive symptoms. These findings affirmed that dynamic metrics of SCN, CN, ATN, and DMN could indicate depressive symptomatology [19, 26, 60].

In our study, subtle changes of dynamic properties in FEDN MDD patients were also recognized. In a previous study, Yan et al. found there was no significant difference in the within-network FC of DMN between FEDN MDD patients and healthy controls using traditional static analysis [8]. However, a subsequent study using a sliding window approach reported robust group differences in temporal variability, average temporal correlation coefficient, and average characteristic temporal path length between FEDN MDD patients and healthy controls [19]. In addition, our results demonstrate that CAP analysis can detect time-varying differences between FEDN MDD patients and recurrent MDD patients in transition probabilities. Such alterations in dynamic properties may allow an early track of illness trajectory and clinical treatment response in MDD patients.

### MDD classification using dynamic metrics

The current diagnosis of MDD is based on Structured Clinical Interview of Diagnostic, such as DSM-IV, HAMD score, or international Classification of Disease. However, such rating scales bring unavoidable decreases in diagnostic reliability since they are measured by patients’ subjective experiences. Thus, more objective tools, such as fMRI [2], are needed for accurate diagnosis and further treatment. In this study, we found dynamic fMRI metrics achieved accuracies of 84.69%, 76.77%, and 88.10% in distinguishing MDD patients, FEDN MDD patients, and recurrent MDD patients from healthy controls, indicating that dynamic functional properties may serve as potential biomarkers for stratifying MDD patients from healthy controls. Our model constructed with SVM and transient networks is comparable to those studies using static FC [12–14, 61], dynamic FC [62–64], the joint of two FCs [62] as input features and is superior to models constructed with structural features [65] in MDD.

### Limitations and future directions

We acknowledge several limitations in the current study. First, the determination of optimal number of *k* (number of clusters) was challenging. Even though we have adopted a series of indices for the evaluation of clustering performance (elbow criteria, SIL, CH, DB, and DVI), we still cannot determine the most optimal *k* value. The frame numbers of fMRI in current study were 240. If *k* increases, there will be fewer frames assigned to a specific CAP, which will lead to loss of clustering stability and dynamic information [66]. CAP cannot reflect the interaction between different networks if the value of *k* is too small. Therefore, a trade-off value of *k* = 7 was chosen in accordance with previous studies [34–36]. Dynamic fMRI analysis with more time points in future studies is suggested for stable clustering and enriched dynamic information in future studies. Second, fMRI with temporal resolution of 2 s might be insufficient to (indirectly) reflect the transient neuronal activity. Future studies could combine fMRI and other neuroimaging modalities, such as electroencephalography and magnetoencephalography to investigate the neuronal correlates of CAPs. Third, head motion can negatively affect fMRI data quality. Despite there being no difference of framewise displacement among each CAP, some de-nosing approaches such as ICA-FIX could be applied after raw data were shared. Finally, we only used dynamic FC for MDD classification here. Although the dynamic properties can achieve a preferable MDD classification, future research can combine static FC features and other modal features such as structural features to achieve better classification performance.

### Conclusions

In conclusion, the present study revealed that MDD patients exhibited aberrant and robust temporal dynamics measured by CAP approach, providing new perspectives for understanding the underlying neural mechanisms in MDD population. The related transient resting-state co-activation networks included activated DMN, SCN, de-activated SCN-CN, de-activated DMN-ATN, and a joint network. Some dynamic properties of these networks were associated with depressive severity. Abnormal fluctuations in deactivated DMN-ATN were also identified in FEDN and recurrent MDD patients. Moreover, temporal dynamics showed predictive value in distinguishing the pooled MDD, FEDN, and recurrent MDD from healthy controls, highlighting temporal dynamics as valuable neuroimaging biomarkers for the diagnosis and future clinical treatment of MDD.

### Supplementary information

SUPPLEMENTAL MATERIAL

## Data Availability

Multi-site MDD dataset is available from Rest-meta-MDD project of DIRECT Consortium upon reasonable request (http://rfmri.org/REST-meta-MDD).

## Code availability

Analysis code in this study is available from the corresponding author (Yanqiu Feng, foree@smu.edu.cn).
