# Supplementary material for: Aberrant resting-state co-activation network dynamics in major depressive disorder
Source: Transl Psychiatry. 2024 Jan 3;14:1. doi: 10.1038/s41398-023-02722-w (PMC10764934; doi:10.1038/s41398-023-02722-w)
Supplement: Supplementary file 1 — SUPPLEMENTAL MATERIAL [file 41398_2023_2722_MOESM1_ESM.docx]

**Supplementary Material**

**1.1 Exclusion criteria.**

1) Subjects had incomplete information on gender, age and education. 2) The ages of subjects were less than 18 or higher than 65. 3) HAMD scores of MDD patients were less than 7. 4) Subjects with excessive head motion in image acquisition (mean frame-wise displacement > 0.2 mm) (1). 5) Subjects had null values of time series extracted from ROIs in post-processing step. 6) The images of subjects had distortion and low quality by visual inspection.

**1.2 Pre-processing of fMRI**

All data were pre-processed using the Data Processing Assistant for Resting-State fMRI (DPARSF) (2). Briefly, slice-timing and motion correction were performed after discarding the first 10 volumes. Then, T1-weighted images were co-registered to functional images and then segmented into gray matter, white matter and cerebrospinal fluid using the Diffeomorphic Anatomical Registration Through Exponentiated Lie Algebra (DARTEL) algorithm. Next, functional images were transformed into Montreal Neurological Institute (MNI) space. Some nuisance variables, including Friston-24 head motion parameters, signal of white matter and cerebrospinal fluid, were removed from the data through linear regression. Finally, spatial smoothing (4 mm full-width at half-maximum Gaussian kernel) and temporal filtering (0.01-0.1 Hz) were applied.

**1.3 Calculation of temporal properties**

1) **dwell time**, measured as the amount of time spent in a given CAP over the whole time series. Longer dwell time means that subjects reside longer consecutive timepoint in a CAP and demonstrate decreased network dynamics. 2) **occurrence rate**, defined as the number occurrences of a given CAP divided by the total number of CAP occurrences. Higher occurrence rate means higher network variability. 3) **transition probability matrix**, calculated as the probability of switching from a certain CAP to another (3, 4). In transition probability matrix, the values of the diagonal matrix represent the probability of switching within a certain CAP, namely persistence probability. Increased transition probability reflects higher network dynamics. 4) **Entropy of Markov Trajectories**, we consider the CAP series as a finite machine. A Markov chain modeling can be derived from these CAP series. We measured the entropy of Markov chain with transition probability matrices. The smaller the entropy is, the more certainty the transition from a CAP to another CAP, which means the transition is less variable and the destination is more accessible. Detailed calculations of entropy of Markov trajectories can be found in **section 1.4**.

**1.4 Entropy of Markov Trajectories**

For a stationary Markov chain, the entropy rate is defined as $H(X)=-\sum_{ij} \mu_{i}P_{ij}\log(P_{ij})$, where $\mu$ is the stationary distribution (probability) solved by $\mu_{j}=\sum_{i} \mu_{i}P_{ij}$, and $P_{ij}$ is the transition probability matrix. Entropy of random trajectory from state $i$ back to state $i$ is defined as $H_{ii}=\frac{H(X)}{\mu_{i}}$, where $\mu_{i}$ is the stationary probability for state $i$. Then matrix $H$ of trajectory from state $i$ to state $j$ is given by $H=K-\tilde{K}+H_{\Delta}$, where $K=(I-P+A)^{-1}(H^{*}-H_{\Delta})$, $I$ is the identity matrix, $P$ is the transition matrix of an irreducible finite state Markov chain, $A$ is the stationary probability with entries $A_{ij}=\mu_{j}$, $H^{*}$ is the first step entropy defined as $H(P_{i})=-\sum_{j} P_{ij}\log(P_{ij})$ and $H_{\Delta}$ is entropy of random trajectory from state $i$ back to state $i$ (diagonal matrix) defined above. $\tilde{K}$ is a matrix defined as $\tilde{K}_{ij}=K_{jj}$. The entropy of Markov trajectories calculations was calculated using the code in the article by Huang et al. in 2020 (Huang et al., 2020). All other three metrics were implemented with custom scripts in MATLAB R2016a (The MathWorks, Inc., Natick, MA, USA).

**1.5 MDD classification using different dynamic properties**

In addition, we also tested the effect of dwell time and occurrence rate, transition probability matrix, and entropy of Markov trajectories on classification performance, respectively. Our analyses revealed accuracies of 74.98% (*P* = 0.001), 80.03% (*P* = 0.001), 77.10% (*P* = 0.001) using features of dwell time and occurrence rate in three groups, while transition probability matrix yielded accuracies of 80.93% (*P* = 0.001), 84.20% (*P* = 0.001), 83.93% (*P* = 0.001) and entropy of Markov trajectories yielded accuracies of 74.54% (*P* = 0.001), 81.24% (*P* = 0.001), 74.76% (*P* = 0.002). The mean AUCs using dwell time and occurrence for the three groups were 0.81 (*P* = 0.002), 0.87 (*P* = 0.001), 0.84 (*P* = 0.001), whereas using transition probability matrix the model reached AUCs of 0.89 (*P* = 0.001), 0.92 (*P* = 0.001), 0.91 (*P* = 0.001). Using features of entropy of Markov trajectories, SVM reached mean AUCs of 0.81 (*P* = 0.001), 0.88 (*P* = 0.001) and 0.84 (*P* = 0.001) in classifying MDD patients (**Table S7-10**).

**1.6 Supplementary Figure**


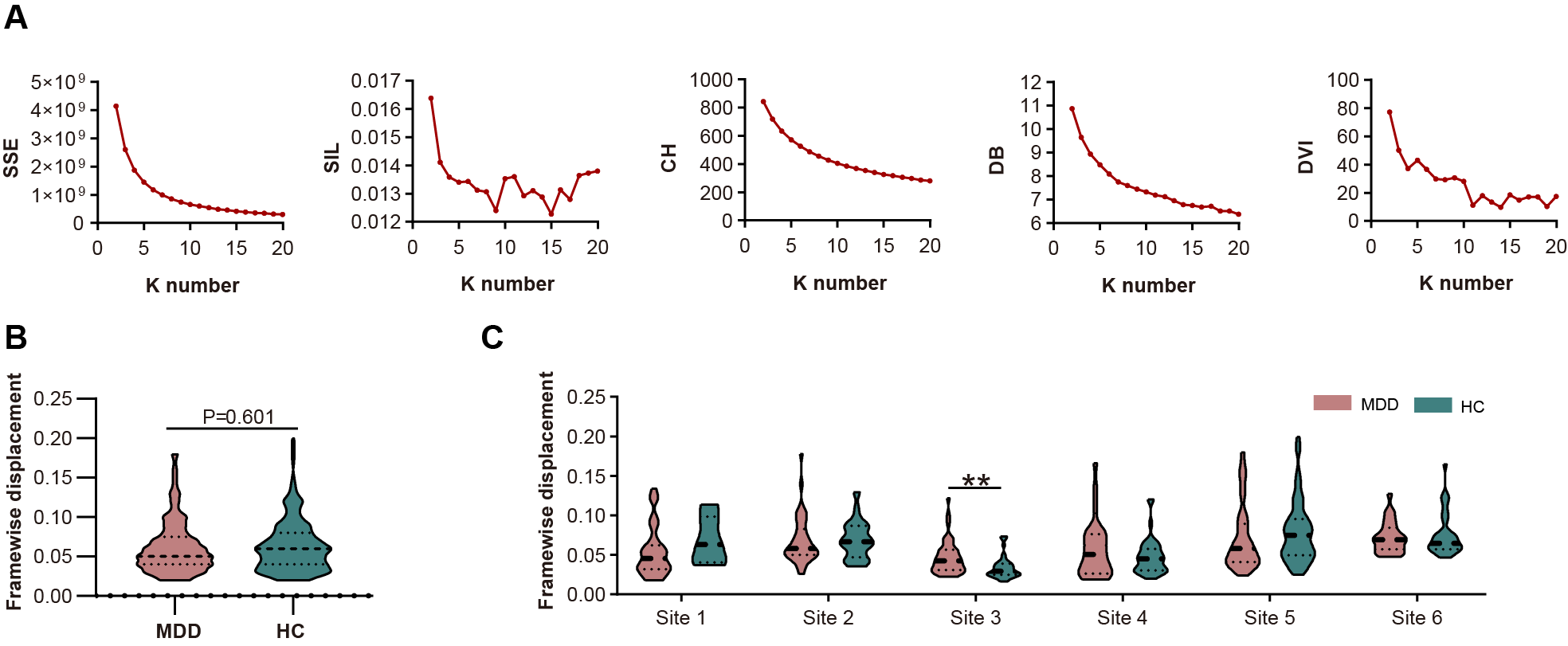


**Figure S1 | (A)** Evaluate the clustering performance using SSE, SIL, CH, DB and DVI. The large values of SIL, CH, DVI, the small value of DB, indicate better clustering performance. **(B)** Frame-wise displacement between MDD patients and healthy controls. **(C)** Frame-wise displacement comparison in each site. Two sample t test with covariates. * *P* < 0.05, ** *P* < 0.01, *** *P* < 0.001, **** *P* < 0.0001. Abbreviations: SSE, elbow criteria; SIL, Silhouette score; SH, Calinski - Harabasz; DB, Davies - Bouldin; DVI, Dunn Validity index.


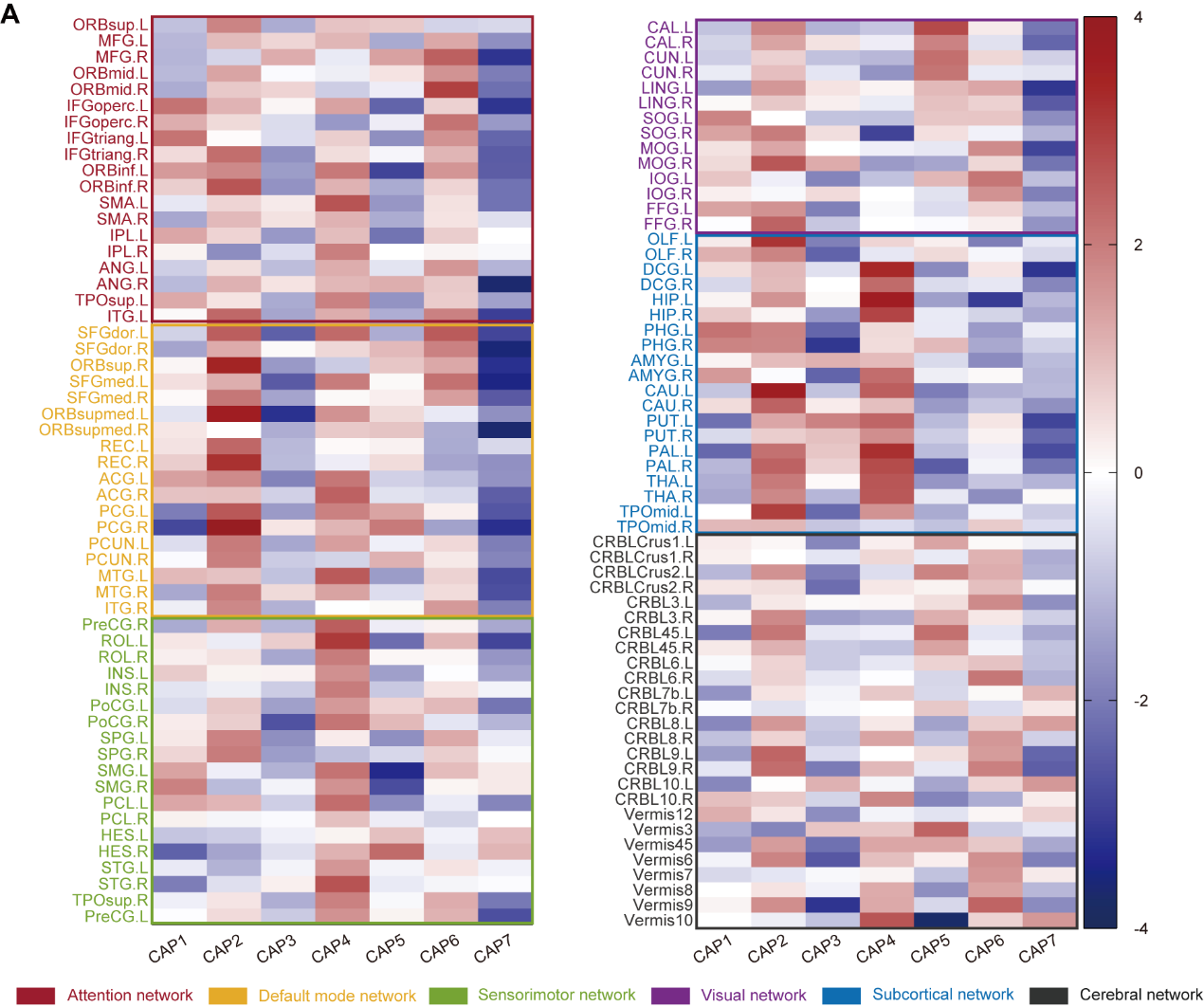


**Figure S2 | (A)** Z statistics of ROIs in each CAP. See **Table S11** for ROI abbreviations.

CAP1 was mainly dominated by SCN (both activated and de-activated, proportion = 30.02%). However, the right posterior cingulate gyrus with the largest absolute *Z* statistic (*Z* = -2.827) was located in the DMN, followed by the right transverse temporal gyri (*Z* = -2.404) belonging to SMN and the left globus pallidus (*Z* = -2.316) belonging to SCN. CAP2 was characterized by positive activation of DMN (proportion = 43.36%). Activated ROIs (*Z* > 3.5) included right superior frontal gyrus (medial orbital part), right posterior cingulate gyrus, left caudate nucleus and left superior frontal gyrus (medial orbital part), etc... CAP3 was the de-activation of SCN (proportion = 35.25%) and CN (proportion = 34.20%). The recurring ROIs (|*Z|* > 3) included left superior frontal gyrus (medial orbital party) belonging to DMN, Lobule IX of vermis belonging to CN and right parahippocampal gyrus belonging to SCN. CAP4 was mainly overlapped with activated SCN (proportion = 42.93%). ROIs with relevant activation (*Z* > 3) included left hippocampus, left middle cingulate, left globus pallidus belonging to SCN and left Rolandic operculum belonging to SMN. CAP5 was a joint network containing CN, ATN, SMN and VN (proportions = 20.85%, 18.51%, 26.48% and 17.91, respectively). Relevant ROIs (|*Z*| > 3) were observed in Lobule X of vermis (nodulus) belonging to CN and left supramarginal gyrus belonging to SMN. CAP6 was mainly dominated by activated ATN (proportion =34.55%), where left hippocampus (*Z* = -3.05) belonging to SCN and right middle frontal gyrus (orbital part) (*Z* = 2.95) belonging to ATN were relevantly activated ROIs. CAP7 corresponded with de-activated DMN (proportion = 32.95%) including regions (|*Z*| > 3.2) of right angular gyrus from ATN, the medial orbital part of right superior frontal gyrus, the dorsolateral of superior frontal gyrus, right posterior cingulate gyrus and orbital part of right superior frontal gyrus from DMN.


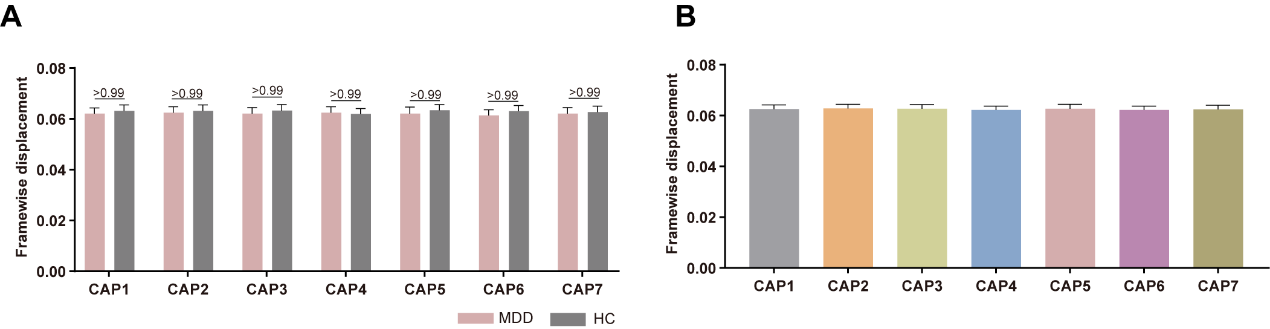


**Figure S3 | (A)** Difference of framewise displacement between MDD and HC in each CAP. Two-way ANOVA with Sidak’s multiple comparison test. **(B)** Difference of framewise displacement between each CAP. One-way ANOVA with Tukey’s multiple comparison test.


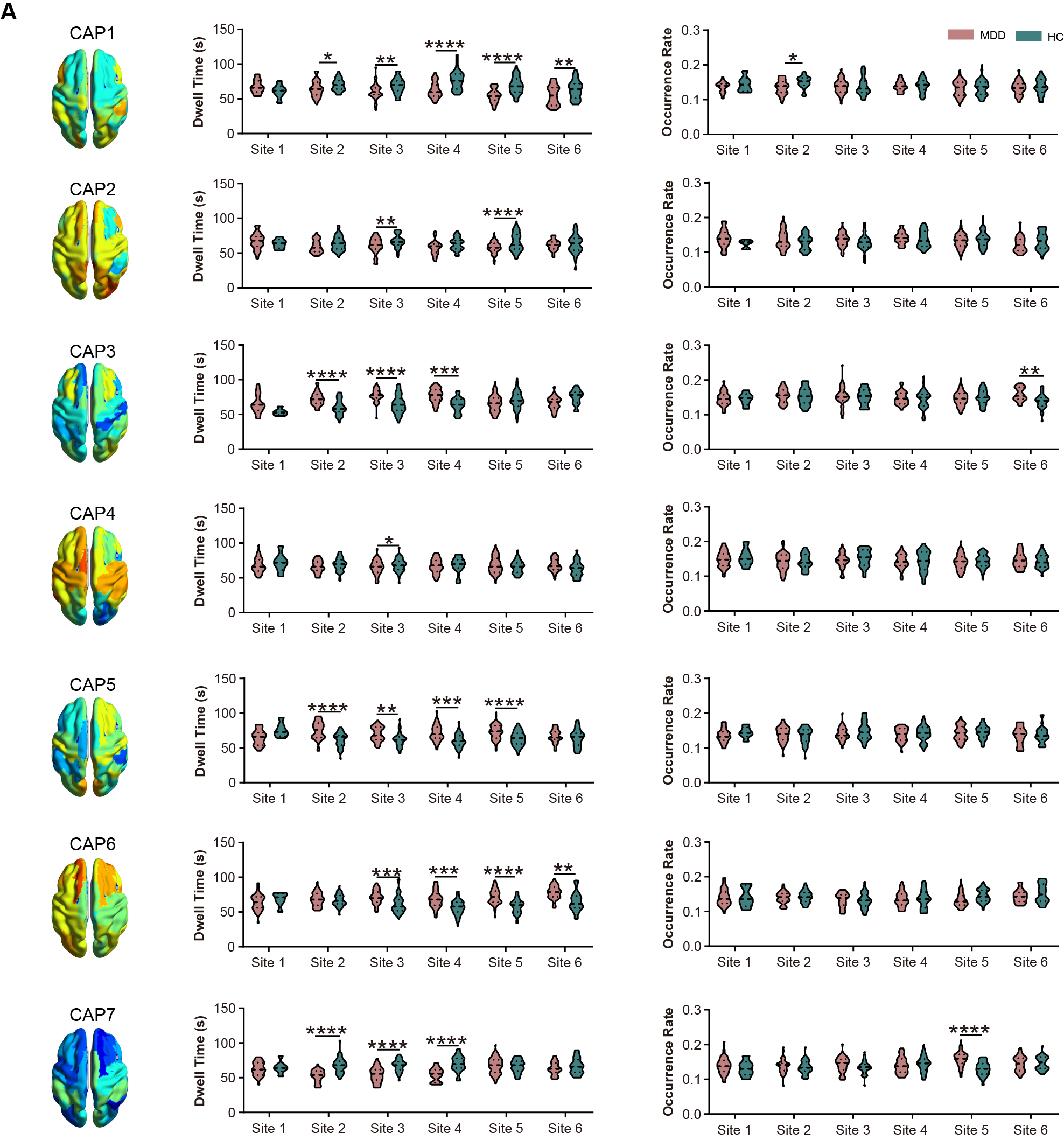


**Figure S4 | (A)** Dwell time and occurrence rate comparison between MDD patients and healthy controls at site-level. Two sample t test with covariates. Multiple comparison with FDR correction. * *P* < 0.05, ** *P* < 0.01, *** *P* < 0.001, **** *P* < 0.0001.


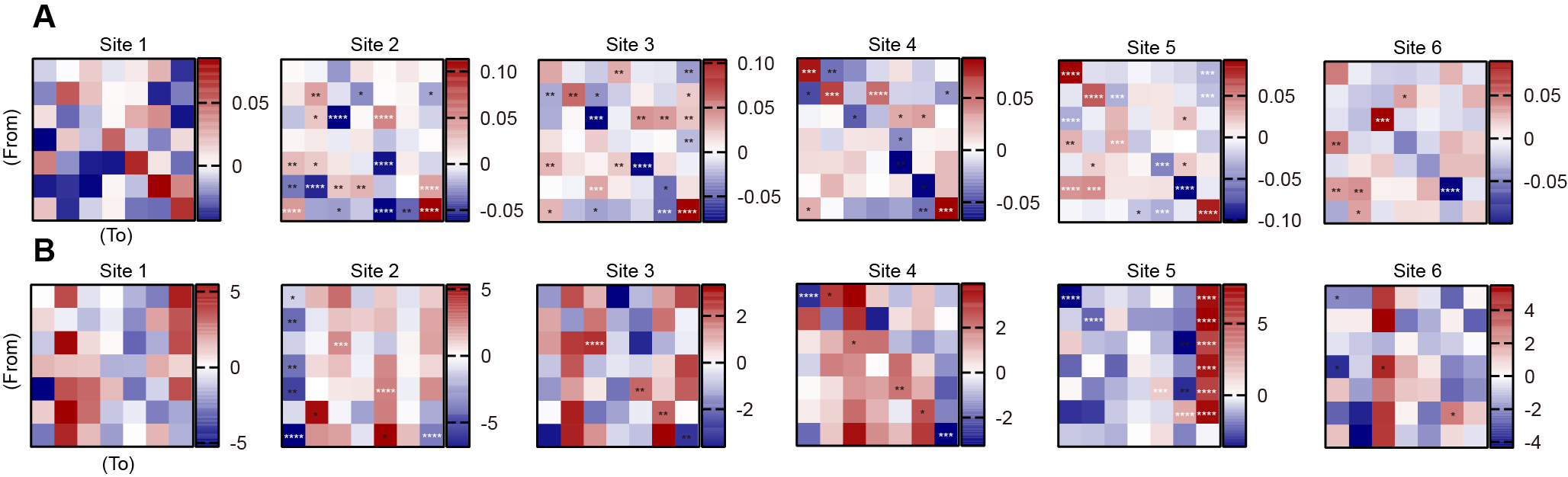


**Figure S5 | (A-B)** Transition probability and entropy of Markov trajectories comparison between MDD patients and healthy controls at site-level.


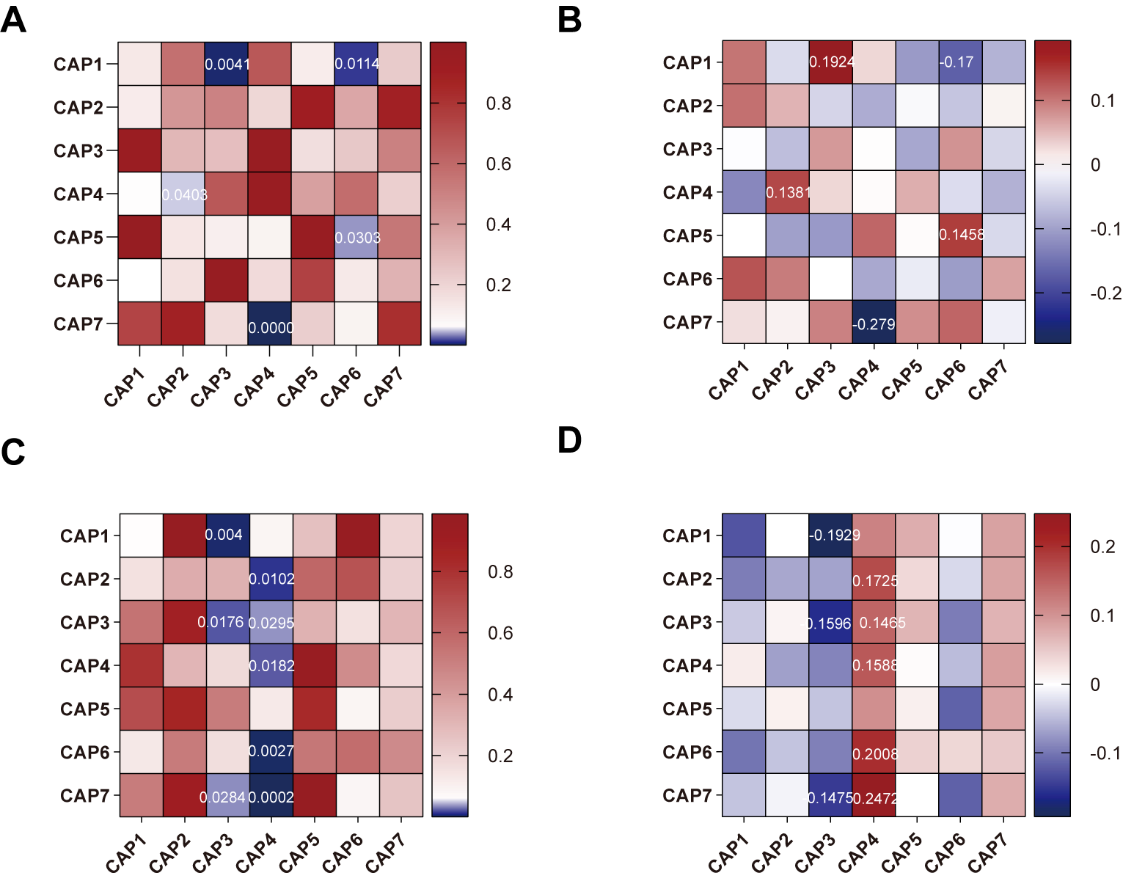


**Figure S6 | (A)** P value of Pearson correlation between transition probabilities and HAMD score. **(B)** R value of Pearson correlation between transition probabilities and HAMD score. **(C)** P value of Pearson correlation between entropy of Markov trajectories and HAMD score. **(D)** R value of Pearson correlation between entropy of Markov trajectories and HAMD score. All MDD patients vs. all healthy controls.


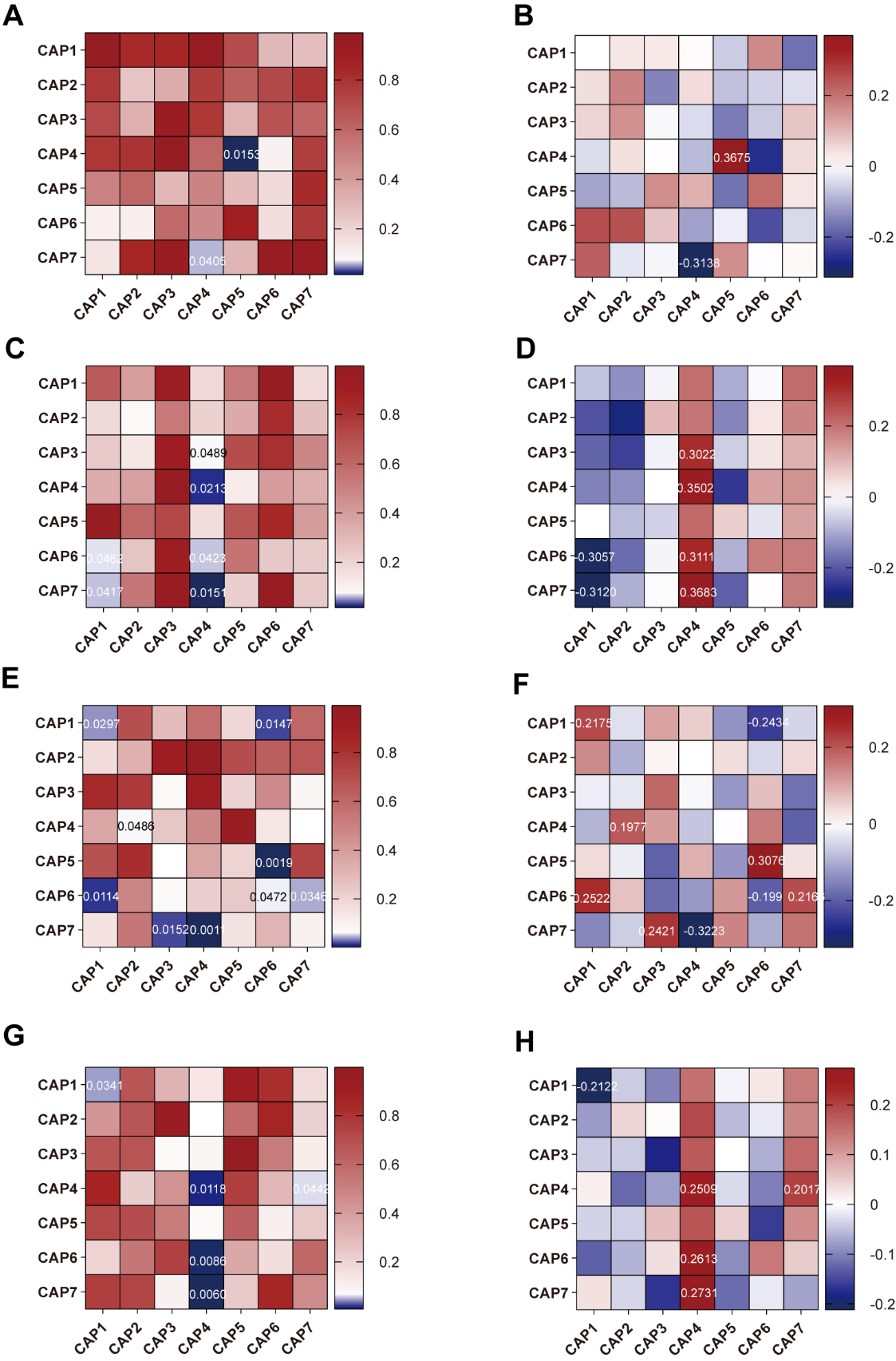


**Figure S7 | (A-B)** P value and R value of Pearson correlation between transition probabilities and HAMD score (FEDN MDD patients vs. healthy controls1). **(C-D)** P value and R value of Pearson correlation between entropy of Markov trajectories and HAMD score (FEDN MDD patients vs. healthy controls1). **(E-F)** P value and R value of Pearson correlation between transition probabilities and HAMD score (recurrent MDD patients vs. healthy controls1). **(G-H)** P value and R value of Pearson correlation between entropy of Markov trajectories and HAMD score (recurrent MDD patients vs. healthy controls1).


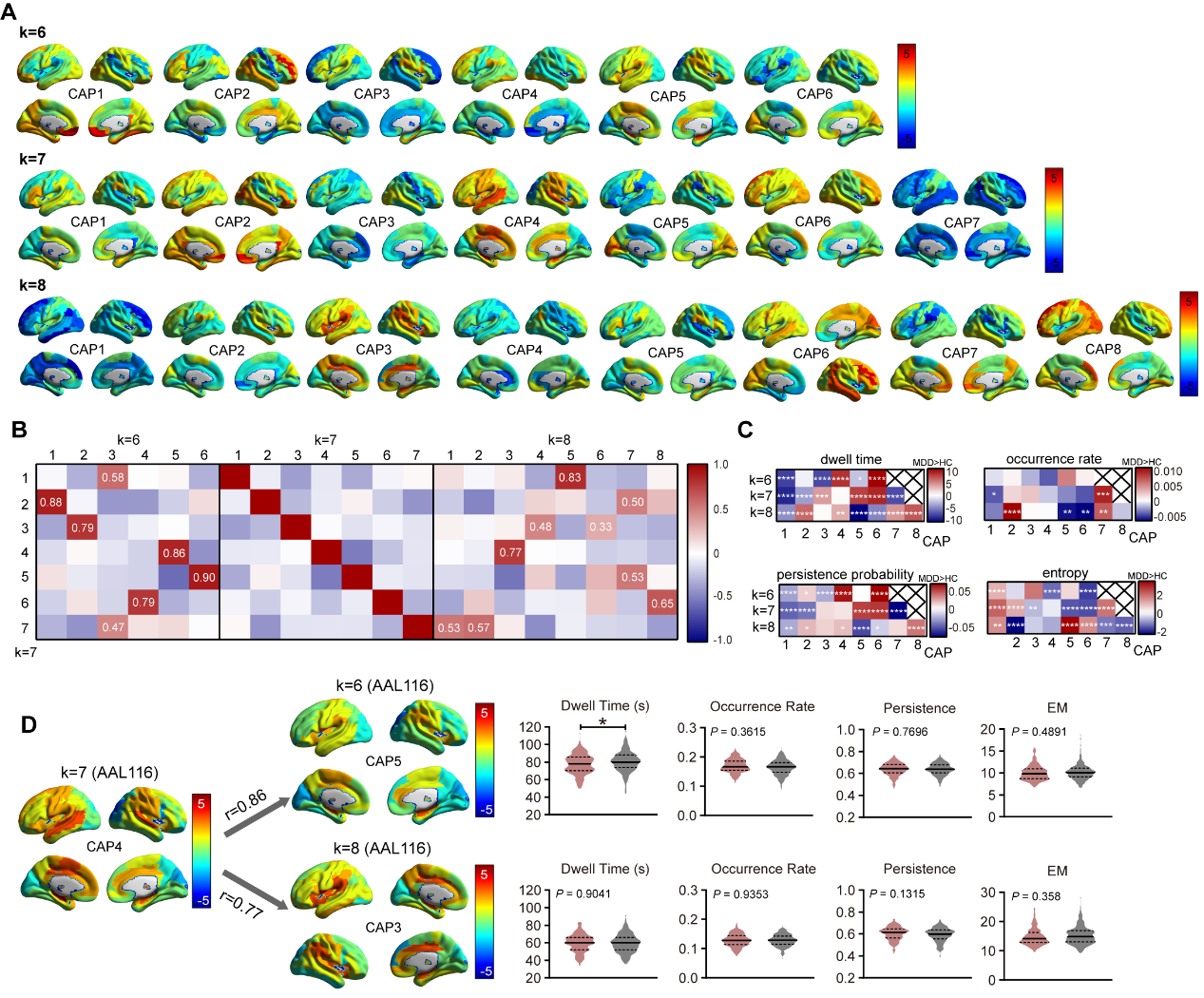


**Figure S8 | (A)** Spatial CAP maps of *k*=6, *k*=7 and *k*=8. **(B)** Spatial similarity between *k* = 6, *k* = *7* and *k* = *8*. **(C)** Group difference on dynamic properties. **(D)** Sine CAP4 has no obvious change in MDD patients, we identified two spatially similar CAPs when *k* = 6 and *k* = *8*. There was no difference on dynamic properties between MDD patients and healthy controls except dwell time of CAP5 when *k* = 6.

**1.7 Supplementary Table**

**Table S1. Site information and data acquisition parameters**

| Site number | Sites (cohorts) | Principal investigators | Data organizer | Sample size | Scanner | TR | TE | Time points |
| --- | --- | --- | --- | --- | --- | --- | --- | --- |
| 1 | Department of Psychiatry, The First  Affiliated Hospital of Chongqing Medical University | Hua-Qing  Meng /  Qing-Hua  Luo | Hai-Tang Qiu | 38 | GE Signa 3T | 2000 | 30 | 240 |
| 2 | Department of Psychosomatics and  Psychiatry, Zhongda Hospital, School of Medicine, Southeast University | Yong-Gui  Yuan | Zheng-Hua Hou / Ying-ying Yin | 100 | Siemens Verio 3.0T MRI | 2000 | 25 | 240 |
| 3 | Department of Psychiatry, The First  Affiliated Hospital of Chongqing Medical University | Li Kuang | Lan Hu | 91 | GE Signa 3T | 2000 | 40 | 240 |
| 4 | Anhui Medical University | Kai Wang | Tong-Jian Bai | 87 | GE Signa 3T | 2000 | 30 | 240 |
| 5 | Beijing Anding Hospital, Capital Medical University | Chuan-Yue  Wang | Qi-Jing  Bo / Feng Li | 156 | Siemens Tim Trio 3T | 2000 | 30 | 240 |
| 6 | Mental Health Center, West China Hospital, Sichuan University | Tao Li | Yi-Ting Zhou | 62 | Philips Achieva 3.0T TX | 2000 | 30 | 240 |

**Table S2. CAP similarities**

| Pearson Correlation | CAP1 | CAP2 | CAP3 | CAP4 | CAP5 | CAP6 | CAP7 |
| --- | --- | --- | --- | --- | --- | --- | --- |
| CAP1 | 1 | -0.0755 | -0.3784 | -0.0913 | -0.3365 | -0.0482 | 0.0274 |
| CAP2 | -0.0755 | 1 | -0.2757 | -0.0863 | 0.0537 | -0.1981 | -0.5230 |
| CAP3 | -0.3784 | -0.2757 | 1 | -0.0191 | -0.1194 | -0.0451 | -0.1134 |
| CAP4 | -0.0913 | -0.0863 | -0.0191 | 1 | -0.5153 | -0.2404 | -0.1060 |
| CAP5 | -0.3365 | 0.0537 | -0.1194 | -0.5153 | 1 | -0.08 | -0.1108 |
| CAP6 | -0.0482 | -0.1981 | -0.0451 | -0.2404 | -0.08 | 1 | -3.004 |
| CAP7 | 0.0274 | -0.5230 | -0.1134 | -0.1060 | -0.1108 | -0.3004 | 1 |

**Table S3. P value of dynamic properties (dwell time, occurrence rate, persistent probability)**

| Group | Dynamic properties | CAP1 | CAP2 | CAP3 | CAP4 | CAP5 | CAP6 | CAP7 |
| --- | --- | --- | --- | --- | --- | --- | --- | --- |
| Healthy controls vs.  MDD | Dwell time | 0.0000 | 0.0000 | 0.0002 | 0.8717 | 0.0000 | 0.0000 | 0.0000 |
|  | Occurrence rate | 0.0274 | 0.5834 | 0.5092 | 0.5834 | 0.4111 | 0.3712 | 0.0001 |
|  | Persistent probability | 0.0000 | 0.0000 | 0.0836 | 0.3384 | 0.0000 | 0.0000 | 0.0000 |
| Healthy controls vs.  FEDN MDD | Dwell time | 0.0001 | 0.3557 | 0.2943 | 0.6971 | 0.0864 | 0.0000 | 0.0094 |
|  | Occurrence rate | 0.7707 | 0.7707 | 0.2727 | 0.5503 | 0.2727 | 0.5503 | 0.6171 |
|  | Persistent probability | 0.0000 | 0.0994 | 0.4205 | 0.2873 | 0.0043 | 0.0043 | 0.0002 |
| Healthy controls vs.  recurrent MDD | Dwell time | 0.0000 | 0.0003 | 0.8393 | 0.1806 | 0.0000 | 0.0000 | 0.0282 |
|  | Occurrence rate | 0.1850 | 0.5749 | 0.7567 | 0.8282 | 0.8282 | 0.0319 | 0.0000 |
|  | Persistent probability | 0.0000 | 0.0000 | 0.0243 | 0.4363 | 0.0001 | 0.0000 | 0.0000 |
| FEDN MDD  Vs.  Recurrent MDD | Dwell time | 0.7793 | 0.1976 | 0.7793 | 0.7842 | 0.1976 | 0.7842 | 0.7793 |
|  | Occurrence rate | 0.2063 | 0.9140 | 0.1926 | 0.3729 | 0.1926 | 0.1926 | 0.1926 |
|  | Persistent probability | 0.8612 | 0.3106 | 0.9960 | 0.5125 | 0.8612 | 0.5125 | 0.5125 |

**Table S4. Correlation statistics between dynamic properties and HAMD scores (dwell time, occurrence rate, persistent probability) with HAMD**

| Group | Dynamic properties | Statistics | CAP1 | CAP2 | CAP3 | CAP4 | CAP5 | CAP6 | CAP7 |
| --- | --- | --- | --- | --- | --- | --- | --- | --- | --- |
| Healthy controls vs.  MDD | Dwell time | r | 0.1118 | 0.0526 | 0.1715 | -0.1882 | -0.0383 | -0.0483 | -0.0752 |
|  |  | *P* | 0.0973 | 0.4369 | 0.0107 | 0.0050 | 0.5714 | 0.4748 | 0.2655 |
|  | Occurrence rate | r | 0.0733 | 0.0228 | 0.1113 | -0.1725 | -0.0176 | 0.0806 | -0.0776 |
|  |  | *P* | 0.2778 | 0.7364 | 0.0988 | 0.0102 | 0.7952 | 0.2325 | 0.2504 |
|  | Persistent probability | r | 0.1023 | 0.0542 | 0.0733 | 0.003 | 0.0023 | -0.1035 | -0.0156 |
|  |  | *P* | 0.1296 | 0.4225 | 0.2778 | 0.9645 | 0.9724 | 0.1250 | 0.8174 |
| Healthy controls vs.  FEDN MDD | Dwell time | r | 0.1096 | 0.2604 | 0.0433 | -0.3183 | 0.0055 | -0.0876 | 0.0061 |
|  |  | *P* | 0.4842 | 0.0917 | 0.7826 | 0.0375 | 0.9719 | 0.5764 | 0.9691 |
|  | Occurrence rate | r | 0.2459 | 0.1186 | -0.0169 | -0.2887 | 0.1228 | -0.0254 | -0.0778 |
|  |  | *P* | 0.3917 | 0.7851 | 0.9146 | 0.3917 | 0.7851 | 0.9146 | 0.8680 |
|  | Persistent probability | r | -0.0024 | 0.1767 | -0.0093 | -0.0815 | -0.1668 | -0.2134 | 0.0060 |
|  |  | *P* | 0.9879 | 0.2569 | 0.9528 | 0.6032 | 0.2852 | 0.1695 | 0.9696 |
| Healthy controls vs.  recurrent MDD | Dwell time | r | 0.1539 | -0.0575 | 0.2391 | -0.2532 | -0.0454 | -0.1064 | 0.0461 |
|  |  | *P* | 0.1264 | 0.5700 | 0.0166 | 0.0110 | 0.6538 | 0.2921 | 0.6491 |
|  | Occurrence rate | r | 0.0671 | 0.0625 | 0.0324 | -0.1864 | 0.0738 | 0.0947 | -0.1150 |
|  |  | *P* | 0.5073 | 0.5370 | 0.7490 | 0.0634 | 0.4657 | 0.3487 | 0.2547 |
|  | Persistent probability | r | 0.2175 | -0.0983 | 0.1788 | -0.0718 | -0.1286 | -0.1989 | 0.1656 |
|  |  | *P* | 0.0298 | 0.3304 | 0.0751 | 0.4780 | 0.2024 | 0.0472 | 0.0996 |

**Table S5. P value of transition probability**

**MDD vs. HC**

**(To)**

|  | CAP1 | CAP2 | CAP3 | CAP4 | CAP5 | CAP6 | CAP7 |
| --- | --- | --- | --- | --- | --- | --- | --- |
| CAP1 |  | 0.0183 | 0.0003 | 0.6235 | 0.1759 | 0.3254 | 0.0001 |
| CAP2 | 0.0053 |  | 0.0000 | 0.2637 | 0.3150 | 0.0251 | 0.0209 |
| CAP3 | 0.0000 | 0.9304 |  | 0.0836 | 0.0000 | 0.0000 | 0.3150 |
| CAP4 | 0.0003 | 0.1294 | 0.0075 |  | 0.0322 | 0.9974 | 0.2464 |
| CAP5 | 0.0117 | 0.0020 | 0.0836 | 0.9720 |  | 0.0003 | 0.3150 |
| CAP6 | 0.0961 | 0.0961 | 0.0014 | 0.0117 | 0.6712 |  | 0.0117 |
| CAP7 | 0.0012 | 0.0674 | 0.0016 | 0.0002 | 0.0000 | 0.0000 |  |

**(From)**

**FEDN MDD vs. HC**

**(To)**

|  | CAP1 | CAP2 | CAP3 | CAP4 | CAP5 | CAP6 | CAP7 |
| --- | --- | --- | --- | --- | --- | --- | --- |
| CAP1 |  | 0.1671 | 0.0424 | 0.1601 | 0.6985 | 0.1904 | 0.0611 |
| CAP2 | 0.1553 |  | 0.1671 | 0.0002 | 0.8623 | 0.1671 | 0.1035 |
| CAP3 | 0.4171 | 0.3263 |  | 0.2873 | 0.1966 | 0.2006 | 0.2416 |
| CAP4 | 0.1152 | 0.2971 | 0.2971 |  | 0.0156 | 0.6835 | 0.1553 |
| CAP5 | 0.5367 | 0.1671 | 0.1901 | 0.1553 |  | 0.0994 | 0.0356 |
| CAP6 | 0.1553 | 0.0994 | 0.1671 | 0.4316 | 0.1553 |  | 0.4205 |
| CAP7 | 0.7187 | 0.6835 | 0.4205 | 0.9967 | 0.6110 | 0.0001 |  |

**(From)**

**Recurrent MDD vs. HC**

**(To)**

|  | CAP1 | CAP2 | CAP3 | CAP4 | CAP5 | CAP6 | CAP7 |
| --- | --- | --- | --- | --- | --- | --- | --- |
| CAP1 |  | 0.0180 | 0.0202 | 0.2868 | 0.1068 | 0.3954 | 0.0057 |
| CAP2 | 0.0029 |  | 0.0002 | 0.0015 | 0.3438 | 0.0488 | 0.0102 |
| CAP3 | 0.0006 | 0.1334 |  | 0.0285 | 0.0548 | 0.0021 | 0.0488 |
| CAP4 | 0.0002 | 0.0516 | 0.0548 |  | 0.2093 | 0.7819 | 0.2657 |
| CAP5 | 0.7461 | 0.1334 | 0.3438 | 0.7010 |  | 0.0063 | 0.2120 |
| CAP6 | 0.0001 | 0.0000 | 0.0952 | 0.5035 | 0.1334 |  | 0.4433 |
| CAP7 | 0.7461 | 0.8451 | 0.2466 | 0.0021 | 0.0001 | 0.0102 |  |

**(From)**

**FEDN MDD vs. Recurrent MDD**

**(To)**

|  | CAP1 | CAP2 | CAP3 | CAP4 | CAP5 | CAP6 | CAP7 |
| --- | --- | --- | --- | --- | --- | --- | --- |
| CAP1 |  | 0.9959 | 0.9676 | 0.5737 | 0.7847 | 0.8612 | 0.9676 |
| CAP2 | 0.7847 |  | 0.7504 | 0.3631 | 0.9960 | 0.8612 | 0.9046 |
| CAP3 | 0.8612 | 0.9960 |  | 0.9960 | 0.9960 | 0.7647 | 0.9959 |
| CAP4 | 0.9451 | 0.9960 | 0.9676 |  | 0.7504 | 0.6094 | 0.2133 |
| CAP5 | 0.4761 | 0.8612 | 0.5877 | 0.6965 |  | 0.9960 | 0.4010 |
| CAP6 | 0.8612 | 0.9406 | 0.0095 | 0.8612 | 0.7504 |  | 0.9960 |
| CAP7 | 0.8612 | 0.7504 | 0.8612 | 0.4010 | 0.3631 | 0.4761 |  |

**(From)**

**Table S6. P value of entropy of Markov trajectories**

**MDD vs. HC**

**(To)**

|  | CAP1 | CAP2 | CAP3 | CAP4 | CAP5 | CAP6 | CAP7 |
| --- | --- | --- | --- | --- | --- | --- | --- |
| CAP1 | 0.0000 | 0.5325 | 0.0241 | 0.4672 | 0.9311 | 0.8218 | 0.0001 |
| CAP2 | 0.5325 | 0.0001 | 0.0040 | 0.2679 | 0.4387 | 0.5881 | 0.0020 |
| CAP3 | 0.8218 | 0.7175 | 0.0088 | 0.6697 | 0.0423 | 0.1271 | 0.0069 |
| CAP4 | 0.0020 | 0.4310 | 0.8091 | 0.5823 | 0.5325 | 0.9311 | 0.0047 |
| CAP5 | 0.0564 | 0.8218 | 0.5297 | 0.8218 | 0.0000 | 0.4987 | 0.0055 |
| CAP6 | 0.0423 | 0.6697 | 0.7195 | 0.6846 | 0.9618 | 0.0000 | 0.0694 |
| CAP7 | 0.0003 | 0.6697 | 0.0672 | 0.8218 | 0.2679 | 0.0482 | 0.0000 |

**(From)**

**FEDN MDD vs. HC**

**(To)**

|  | CAP1 | CAP2 | CAP3 | CAP4 | CAP5 | CAP6 | CAP7 |
| --- | --- | --- | --- | --- | --- | --- | --- |
| CAP1 | 0.0001 | 0.8844 | 0.3250 | 0.8494 | 0.1765 | 0.5135 | 0.4514 |
| CAP2 | 0.7096 | 0.4913 | 0.2158 | 0.1288 | 0.1765 | 0.2158 | 0.8191 |
| CAP3 | 0.8191 | 0.8844 | 0.7096 | 0.9414 | 0.1418 | 0.8191 | 0.7096 |
| CAP4 | 0.7096 | 0.8191 | 0.7096 | 0.8191 | 0.9010 | 0.6519 | 0.8191 |
| CAP5 | 0.7096 | 0.8191 | 0.6698 | 0.8191 | 0.2293 | 0.8191 | 0.8191 |
| CAP6 | 0.8191 | 0.8191 | 0.1765 | 0.7096 | 0.1765 | 0.0020 | 0.8191 |
| CAP7 | 0.9195 | 0.8191 | 0.4321 | 0.7096 | 0.2813 | 0.0201 | 0.0290 |

**(From)**

**Recurrent MDD vs. HC**

**(To)**

|  | CAP1 | CAP2 | CAP3 | CAP4 | CAP5 | CAP6 | CAP7 |
| --- | --- | --- | --- | --- | --- | --- | --- |
| CAP1 | 0.0000 | 0.8164 | 0.2167 | 0.7344 | 0.5348 | 0.2838 | 0.0002 |
| CAP2 | 0.8970 | 0.0005 | 0.0221 | 0.3264 | 0.6156 | 0.7376 | 0.0002 |
| CAP3 | 0.9087 | 0.7376 | 0.4223 | 0.2706 | 0.7376 | 0.0092 | 0.0012 |
| CAP4 | 0.0092 | 0.9113 | 0.7376 | 0.2167 | 0.5348 | 0.6776 | 0.0012 |
| CAP5 | 0.4948 | 0.3657 | 0.5157 | 0.5348 | 0.0002 | 0.0912 | 0.0084 |
| CAP6 | 0.0131 | 0.1205 | 0.4727 | 0.4948 | 0.9087 | 0.0000 | 0.0006 |
| CAP7 | 0.2167 | 0.4697 | 0.4223 | 0.4348 | 0.2387 | 0.9087 | 0.0111 |

**(From)**

**FEDN MDD vs. Recurrent MDD**

**(To)**

|  | CAP1 | CAP2 | CAP3 | CAP4 | CAP5 | CAP6 | CAP7 |
| --- | --- | --- | --- | --- | --- | --- | --- |
| CAP1 | 0.7980 | 0.9119 | 0.8682 | 0.8694 | 0.1582 | 0.2815 | 0.3918 |
| CAP2 | 0.4130 | 0.1582 | 0.8682 | 0.3189 | 0.2739 | 0.3918 | 0.3918 |
| CAP3 | 0.5282 | 0.8694 | 0.5043 | 0.5043 | 0.1590 | 0.2011 | 0.3918 |
| CAP4 | 0.2739 | 0.8694 | 0.8369 | 0.8682 | 0.5043 | 0.5043 | 0.1582 |
| CAP5 | 0.2011 | 0.8694 | 0.9159 | 0.8694 | 0.4130 | 0.3918 | 0.1677 |
| CAP6 | 0.2980 | 0.8694 | 0.2011 | 0.3918 | 0.1590 | 0.8694 | 0.2739 |
| CAP7 | 0.2739 | 0.9453 | 0.5043 | 0.3918 | 0.1582 | 0.1582 | 0.8694 |

**(From)**

**Table S7. SVM performance in distinguishing MDD patients from healthy controls using different features.**

| k value | Group | AUC / P_permutation_ | Accuracy / P_permutation_ | Sensitivity | Specificity | F1 score | PPV | NPV |
| --- | --- | --- | --- | --- | --- | --- | --- | --- |
| k = 6 | All pooled | 0.8911 / 0.001 | 0.8118 / 0.001 | 0.7783 | 0.8463 | 0.8064 | 0.8398 | 0.7910 |
|  | DT & Occ | 0.8091 / 0.002 | 0.7498 / 0.001 | 0.7372 | 0.7626 | 0.7495 | 0.7679 | 0.7382 |
|  | TP | 0.8913 / 0.001 | 0.8093 / 0.001 | 0.8097 | 0.8093 | 0.8108 | 0.8144 | 0.8079 |
|  | EM | 0.8149 / 0.001 | 0.7454 / 0.001 | 0.7462 | 0.7442 | 0.7495 | 0.7594 | 0.7391 |
| k = 7 | All pooled | 0.9265 / 0.001 | 0.8469 / 0.001 | 0.8289 | 0.8660 | 0.8459 | 0.8681 | 0.8334 |
|  | DT & Occ | 0.8711 / 0.001 | 0.8003 / 0.001 | 0.8140 | 0.7864 | 0.8044 | 0.7980 | 0.8075 |
|  | TP | 0.9213 / 0.001 | 0.8420 / 0.001 | 0.8148 | 0.8703 | 0.8396 | 0.8674 | 0.8209 |
|  | EM | 0.8830 / 0.001 | 0.8124 / 0.001 | 0.7923 | 0.8333 | 0.8101 | 0.8330 | 0.7989 |
| k = 8 | All pooled | 0.9263 / 0.001 | 0.8463 / 0.001 | 0.8547 | 0.8374 | 0.8481 | 0.8492 | 0.8552 |
|  | DT & Occ | 0.8386 / 0.001 | 0.7710 / 0.001 | 0.7964 | 0.7448 | 0.7790 | 0.7649 | 0.7822 |
|  | TP | 0.9121 / 0.001 | 0.8393 / 0.001 | 0.8777 | 0.7996 | 0.8481 | 0.8252 | 0.8660 |
|  | EM | 0.8372 / 0.001 | 0.7476 / 0.002 | 0.7462 | 0.7494 | 0.7498 | 0.7672 | 0.7475 |

Abbreviations: DT, dwell time; Occ, occurrence rate; TP, transition probability; EM, entropy of Markov trajectories.

**Table S8. SVM performance in distinguishing FEDN MDD patients from healthy controls using different features.**

| k value | Group | AUC / P_permutation_ | Accuracy / P_permutation_ | Sensitivity | Specificity | F1 score | PPV | NPV |
| --- | --- | --- | --- | --- | --- | --- | --- | --- |
| k = 6 | All pooled | 0.8383 / 0.0230 | 0.7522 / 0.0430 | 0.7650 | 0.7489 | 0.6031 | 0.5127 | 0.9129 |
|  | DT & Occ | 0.6916 / 0.1558 | 0.6844 / 0.0919 | 0.5850 | 0.7176 | 0.4761 | 0.4127 | 0.8429 |
|  | TP | 0.8291 / 0.0450 | 0.7296 / 0.1009 | 0.5950 | 0.7764 | 0.4728 | 0.4513 | 0.8796 |
|  | EM | 0.5744 / 0.3297 | 0.6216 / 0.2348 | 0.4200 | 0.6863 | 0.3441 | 0.3027 | 0.7901 |
| k = 7 | All pooled | 0.8222 / 0.0390 | 0.7677 / 0.0170 | 0.7300 | 0.7808 | 0.5982 | 0.5244 | 0.9048 |
|  | DT & Occ | 0.7527 / 0.0789 | 0.6916 / 0.1059 | 0.6750 | 0.6962 | 0.5072 | 0.4093 | 0.8750 |
|  | TP | 0.8327 / 0.0410 | 0.7049 / 0.1359 | 0.6600 | 0.7209 | 0.5061 | 0.4267 | 0.8780 |
|  | EM | 0.7421 / 0.0809 | 0.6887 / 0.0809 | 0.6150 | 0.7132 | 0.4945 | 0.4196 | 0.8494 |
| k = 8 | All pooled | 0.8160 / 0.0330 | 0.7413 / 0.0440 | 0.7200 | 0.7473 | 0.5829 | 0.5161 | 0.8933 |
|  | DT & Occ | 0.7321 / 0.1149 | 0.6303 / 0.2168 | 0.7200 | 0.6000 | 0.4927 | 0.3793 | 0.8658 |
|  | TP | 0.7392 / 0.0979 | 0.6411 / 0.2368 | 0.7100 | 0.6165 | 0.4837 | 0.3703 | 0.8801 |
|  | EM | 0.8119 / 0.0529 | 0.7131 / 0.0659 | 0.7500 | 0.7022 | 0.5595 | 0.4630 | 0.9015 |

**Table S9. SVM performance in distinguishing recurrent MDD patients from healthy controls using different features.**

| k value | Group | AUC / P_permutation_ | Accuracy / P_permutation_ | Sensitivity | Specificity | F1 score | PPV | NPV |
| --- | --- | --- | --- | --- | --- | --- | --- | --- |
| k = 6 | All pooled | 0.9320 / 0.0010 | 0.8632 / 0.001 | 0.8600 | 0.8665 | 0.8416 | 0.8411 | 0.8984 |
|  | DT & Occ | 0.8664 / 0.0020 | 0.7824 / 0.0140 | 0.8200 | 0.7560 | 0.7550 | 0.7214 | 0.8629 |
|  | TP | 0.9301 / 0.0010 | 0.8848 / 0.0010 | 0.8600 | 0.9038 | 0.8602 | 0.8767 | 0.9048 |
|  | EM | 0.8667 / 0.0040 | 0.7572 / 0.0070 | 0.8000 | 0.7264 | 0.7333 | 0.6888 | 0.8408 |
| k = 7 | All pooled | 0.9171 / 0.0010 | 0.8810 / 0.0010 | 0.8600 | 0.8967 | 0.8601 | 0.8666 | 0.8990 |
|  | DT & Occ | 0.9076 / 0.0030 | 0.8297 / 0.0010 | 0.8400 | 0.8220 | 0.8081 | 0.7832 | 0.8758 |
|  | TP | 0.9128 / 0.0010 | 0.8388 / 0.0020 | 0.8600 | 0.8231 | 0.8183 | 0.7861 | 0.8944 |
|  | EM | 0.8977 0.0010 | 0.8301 / 0.0010 | 0.8300 | 0.8302 | 0.8087 | 0.7961 | 0.8679 |
| k = 8 | All pooled | 0.8938 / 0.0030 | 0.8007 / 0.0020 | 0.8000 | 0.8016 | 0.7707 | 0.7544 | 0.8523 |
|  | DT & Occ | 0.7866 / 0.0160 | 0.7201 / 0.0190 | 0.7700 | 0.6819 | 0.6985 | 0.6431 | 0.8088 |
|  | TP | 0.8857 / 0.0020 | 0.8091 / 0.0030 | 0.7600 | 0.8456 | 0.7742 | 0.8046 | 0.8267 |
|  | EM | 0.7917 / 0.0100 | 0.7203 / 0.0250 | 0.7300 | 0.7126 | 0.6860 | 0.6552 | 0.7904 |

**Table S10. SVM performance in distinguishing FEDN MDD patients from recurrent MDD patients using all dynamic features.**

| k value | Group | AUC / P_permutation_ | Accuracy / P_permutation_ | Sensitivity | Specificity | F1 score | PPV | NPV |
| --- | --- | --- | --- | --- | --- | --- | --- | --- |
| k = 7 | All pooled | 0.7090 / 0.1479 | 0.7214 / 0.0749 | 0.6800 | 0.7400 | 0.5606 | 0.4928 | 0.8612 |
| k = 8 | All pooled | 0.6890 / 0.1309 | 0.7048 / 0.1678 | 0.5000 | 0.7900 | 0.4933 | 0.5481 | 0.7952 |

**Table S11. ROIs’ full names, abbreviations and corresponding functional networks.**

| **Labels** | **Regions** | **Abbr.** | **Functional network** | **Labels** | **Regions** | **Abbr.** | **Functionalnetwork** |
| --- | --- | --- | --- | --- | --- | --- | --- |
| 1 | Precental gyrus | PreCG.L | sensorimotor | 59 | Superior parietal gyrus | SPG.L | sensorimotor |
| 2 | Precental gyrus | PreCG.R | sensorimotor | 60 | Superior parietal gyrus | SPG.R | sensorimotor |
| 3 | Superior frontal gyrus, dorsolateral | SFGdor.L | DMN | 61 | Inferior parietal, but supramarginal and angular gyri | IPL.L | Attention |
| 4 | Superior frontal gyrus, dorsolateral | SFGdor.R | DMN | 62 | Inferior parietal, but supramarginal and angular gyri | IPL.R | Attention |
| 5 | Superior frontal gyrus, orbital part | ORBsup.L | Attention | 63 | Supramarginal gyrus | SMG.L | sensorimotor |
| 6 | Superior frontal gyrus, orbital part | ORBsup.R | DMN | 64 | Supramarginal gyrus | SMG.R | sensorimotor |
| 7 | Middle frontal gyrus | MFG.L | Attention | 65 | Angular gyrus | ANG.L | Attention |
| 8 | Middle frontal gyrus | MFG.R | Attention | 66 | Angular gyrus | ANG.R | Attention |
| 9 | Middle frontal gyrus, orbital part | ORBmid.L | Attention | 67 | Precuneus | PCUN.L | DMN |
| 10 | Middle frontal gyrus, orbital part | ORBmid.R | Attention | 68 | Precuneus | PCUN.R | DMN |
| 11 | Inferior frontal gyrus, opercular part | IFGoperc.L | Attention | 69 | Paracentral lobule | PCL.L | sensorimotor |
| 12 | Inferior frontal gyrus, opercular part | IFGoperc.R | Attention | 70 | Paracentral lobule | PCL.R | sensorimotor |
| 13 | Inferior frontal gyrus, triangular part | IFGtriang.L | Attention | 71 | Caudate nucleus | CAU.L | Subcortical |
| 14 | Inferior frontal gyrus, triangular part | IFGtriang.R | Attention | 72 | Caudate nucleus | CAU.R | Subcortical |
| 15 | Inferior frontal gyrus, orbital part | ORBinf.L | Attention | 73 | Lenticular nucleus, putamen | PUT.L | Subcortical |
| 16 | Inferior frontal gyrus, orbital part | ORBinf.R | Attention | 74 | Lenticular nucleus, putamen | PUT.R | Subcortical |
| 17 | Rolandic operculum | ROL.L | sensorimotor | 75 | Lenticular nucleus, pallidum | PAL.L | Subcortical |
| 18 | Rolandic operculum | ROL.R | sensorimotor | 76 | Lenticular nucleus, pallidum | PAL.R | Subcortical |
| 19 | Supplementary motor area | SMA.L | Attention | 77 | Thalamus | THA.L | Subcortical |
| 20 | Supplementary motor area | SMA.R | Attention | 78 | Thalamus | THA.R | Subcortical |
| 21 | Olfactory cortex | OLF.L | Subcortical | 79 | Heschl gyrus | HES.L | sensorimotor |
| 22 | Olfactory cortex | OLF.R | Subcortical | 80 | Heschl gyrus | HES.R | sensorimotor |
| 23 | Superior frontal gyrus, medial | SFGmed.L | DMN | 81 | Superior temporal gyrus | STG.L | sensorimotor |
| 24 | Superior frontal gyrus, medial | SFGmed.R | DMN | 82 | Superior temporal gyrus | STG.R | sensorimotor |
| 25 | Superior frontal gyrus, medial orbital | ORBsupmed.L | DMN | 83 | Temporal pole: superior temporal gyrus | TPOsup.L | Attention |
| 26 | Superior frontal gyrus, medial orbital | ORBsupmed.R | DMN | 84 | Temporal pole: superior temporal gyrus | TPOsup.R | sensorimotor |
| 27 | Gyrus rectus | REC.L | DMN | 85 | Middle temporal gyrus | MTG.L | DMN |
| 28 | Gyrus rectus | REC.R | DMN | 86 | Middle temporal gyrus | MTG.R | DMN |
| 29 | Insula | INS.L | sensorimotor | 87 | Temporal pole: middle temporal gyrus | TPOmid.L | Subcortical |
| 30 | Insula | INS.R | sensorimotor | 88 | Temporal pole: middle temporal gyrus | TPOmid.R | Subcortical |
| 31 | Anterior cingulate and paracingulate gyri | ACG.L | DMN | 89 | Inferior temporal gyrus | ITG.L | Attention |
| 32 | Anterior cingulate and paracingulate gyri | ACG.R | DMN | 90 | Inferior temporal gyrus | ITG.R | DMN |
| 33 | Median cingulate and paracingulate gyri | DCG.L | Subcortical | 91 | Left crus I of cerebellar hemisphere | CRBLCrus1.L | Cerebral |
| 34 | Median cingulate and paracingulate gyri | DCG.R | Subcortical | 92 | Right crus I of cerebellar hemisphere | CRBLCrus1.R | Cerebral |
| 35 | Posterior cingulate gyrus | PCG.L | DMN | 93 | Left crus II of cerebellar hemisphere | CRBLCrus2.L | Cerebral |
| 36 | Posterior cingulate gyrus | PCG.R | DMN | 94 | Right crus II of cerebellar hemisphere | CRBLCrus2.R | Cerebral |
| 37 | Hippocampus | HIP.L | Subcortical | 95 | Left Lobule III of cerebellar hemisphere | CRBL3.L | Cerebral |
| 38 | Hippocampus | HIP.R | Subcortical | 96 | Right Lobule III of cerebellar hemisphere | CRBL3.R | Cerebral |
| 39 | Parahippocampal gyrus | PHG.L | Subcortical | 97 | Left lobule IV, V of cerebellar hemisphere | CRBL45.L | Cerebral |
| 40 | Parahippocampal gyrus | PHG.R | Subcortical | 98 | Right lobule IV, V of cerebellar hemisphere | CRBL45.R | Cerebral |
| 41 | Amygdala | AMYG.L | Subcortical | 99 | Left Lobule VI of cerebellar hemisphere | CRBL6.L | Cerebral |
| 42 | Amygdala | AMYG.R | Subcortical | 100 | Right Lobule VI of cerebellar hemisphere | CRBL6.R | Cerebral |
| 43 | Calcarine fissure and surrounding cortex | CAL.L | Visual | 101 | Left lobule VIIB of cerebellar hemisphere | CRBL7b.L | Cerebral |
| 44 | Calcarine fissure and surrounding cortex | CAL.R | Visual | 102 | Right lobule VIIB of cerebellar hemisphere | CRBL7b.R | Cerebral |
| 45 | Cuneus | CUN.L | Visual | 103 | Left lobule VIII of cerebellar hemisphere | CRBL8.L | Cerebral |
| 46 | Cuneus | CUN.R | Visual | 104 | Right lobule VIII of cerebellar hemisphere | CRBL8.R | Cerebral |
| 47 | Lingual gyrus | LING.L | Visual | 105 | Left lobule IX of cerebellar hemisphere | CRBL9.L | Cerebral |
| 48 | Lingual gyrus | LING.R | Visual | 106 | Right lobule IX of cerebellar hemisphere | CRBL9.R | Cerebral |
| 49 | Superior occipital gyrus | SOG.L | Visual | 107 | Left lobule X of cerebellar hemisphere (flocculus) | CRBL10.L | Cerebral |
| 50 | Superior occipital gyrus | SOG.R | Visual | 108 | Right lobule X of cerebellar hemisphere (flocculus) | CRBL10.R | Cerebral |
| 51 | Middle occipital gyrus | MOG.L | Visual | 109 | Lobule I, II of vermis | Vermis12 | Cerebral |
| 52 | Middle occipital gyrus | MOG.R | Visual | 110 | Lobule III of vermis | Vermis3 | Cerebral |
| 53 | Inferior occipital gyrus | IOG.L | Visual | 111 | Lobule IV, V of vermis | Vermis45 | Cerebral |
| 54 | Inferior occipital gyrus | IOG.R | Visual | 112 | Lobule VI of vermis | Vermis6 | Cerebral |
| 55 | Fusiform gyrus | FFG.L | Visual | 113 | Lobule VII of vermis | Vermis7 | Cerebral |
| 56 | Fusiform gyrus | FFG.R | Visual | 114 | Lobule VIII of vermis | Vermis8 | Cerebral |
| 57 | Postcentral gyrus | PoCG.L | sensorimotor | 115 | Lobule IX of vermis | Vermis9 | Cerebral |
| 58 | Postcentral gyrus | PoCG.R | sensorimotor | 116 | Lobule X of vermis (nodulus) | Vermis10 | Cerebral |

**Reference**

Huang, Z., Zhang, J., Wu, J., Mashour, G.A., Hudetz, A.G., 2020. Temporal circuit of macroscale dynamic brain activity supports human consciousness. Science advances 6, eaaz0087.
